# The atypical *KRAS* ^Q22K^ mutation directs TGF‐β response towards partial epithelial‐to‐mesenchymal transition in patient‐derived colorectal cancer tumoroids

**DOI:** 10.1002/1878-0261.70014

**Published:** 2025-03-11

**Authors:** Theresia Mair, Philip König, Milena Mijović, Jessica Kalla, Anil Baskan, Loan Tran, Kristina Draganić, Pedro Morata Saldaña, Carlos Uziel Pérez Malla, Janette Pfneissl, Andreas Tiefenbacher, Julijan Kabiljo, Velina S. Atanasova, Lisa Wozelka‐Oltjan, Leonhard Müllauer, Michael Bergmann, Raheleh Sheibani‐Tezerji, Gerda Egger

**Affiliations:** ^1^ Department of Pathology Medical University of Vienna Austria; ^2^ Ludwig Boltzmann Institute Applied Diagnostics Austria; ^3^ Department of General Surgery, Division of Visceral Surgery Medical University of Vienna Austria; ^4^ Institute of Medical Genetics Medical University of Vienna Austria; ^5^ Comprehensive Cancer Center Medical University of Vienna Austria

**Keywords:** colorectal cancer, EMT, *KRAS* mutations, organoids, patient‐derived tumoroids, TGF‐β

## Abstract

Transforming growth factor beta (TGF‐β) exhibits complex and context‐dependent cellular responses. While it mostly induces tumor‐suppressive effects in early stages of tumorigenesis, tumor‐promoting properties are evident in advanced disease. This TGF‐β duality is still not fully understood, and whether TGF‐β supports invasion and metastasis by influencing cancer cells directly, or rather through the stromal tumor compartment, remains a matter of debate. Here, we utilized a library of colorectal cancer (CRC) patient‐derived tumoroids (PDTs), representing a spectrum of tumor stages, to study cancer cell‐specific responses to TGF‐β. Using conditions allowing for the differentiation of PDTs, we observed TGF‐β‐induced tumor‐suppressive effects in early‐stage tumoroids, whereas more advanced tumoroids were less sensitive to the treatment. Notably, one tumoroid line harboring an atypical *KRAS* ^Q22K^ mutation underwent partial epithelial‐to‐mesenchymal transition (EMT), which was associated with morphological changes and increased invasiveness. On a molecular level, this was accompanied by elevated expression of mesenchymal genes, as well as deregulation of pathways associated with matrix remodeling and cell adhesion. Our results suggest that tumor cell‐intrinsic responses to TGF‐β are critical in determining its tumor‐suppressive or tumor‐promoting effects.

## Graphical abstract

TGF‐β has a complex role in cancer, exhibiting both tumor‐suppressive and tumor‐promoting properties. Using a series of differentiated tumoroids, derived from different stages and mutational background of colorectal cancer patients, we replicate this duality of TGF‐β *in vitro.* Notably, the atypical but highly aggressive *KRAS* ^*Q22K*^ mutation rendered early‐stage tumoroids susceptible to TGF‐β treatment and induced partial EMT and increased invasion.

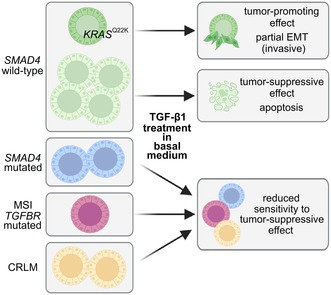

**Abbreviations**
ANOVA: analysis of variance
BSA: bovine serum albumin
CAFs: cancer‐associated fibroblasts
CMS: consensus molecular subtypes
CNET: Gene‐Concept Network
CRC: colorectal cancer
CRLM: colorectal liver metastasis
DE: differential gene expression
DEG: differentially expressed gene
ECM: extracellular matrix
EMT: epithelial‐to‐mesenchymal transition
f: female
FCS: fetal calf serum
FFPE: formalin‐fixed paraffin‐embedded
GSEA: gene set enrichment analysis
H&E: hematoxylin and eosin
IF: Immunofluorescence
LFC: log_2_ fold change
m: male
MSI: microsatellite instable
MSigDB: Molecular Signatures Database
MSS: microsatellite stable
PCA: principal component analysis
PDTs: patient‐derived tumoroids
PFA: paraformaldehyde
qRT‐PCR: quantitative reverse‐transcription PCR
RT: room temperature
SD: standard deviation
TFs: transcription factors
TGF‐β: Transforming growth factor beta
VST: variance stabilizing transformation

## 1 Introduction

The transforming growth factor beta (TGF‐β) family of proteins includes various cytokines and growth factors, which are crucial for tissue integrity during development, homeostasis, and repair processes [1]. TGF‐β signaling affects multiple cell types and exhibits divergent roles either promoting or inhibiting cell proliferation, modulating cellular plasticity, remodeling the extracellular matrix (ECM), and regulating immune tolerance. Canonical TGF‐β signaling is initiated by the activation of latent TGF‐β and subsequent binding to hetero‐dimeric TGF‐β receptor complexes. This results in phosphorylation and activation of SMAD2/3 transcription factors (TFs), which subsequently bind to SMAD4. The SMAD‐complex translocation to the nucleus eventually facilitates transcriptional activation of target genes in a context‐dependent manner through interaction with lineage‐specific or signal‐dependent TFs [1].

In healthy colon tissue, TGF‐β modulates the differentiation and proliferation of epithelial cells and controls inflammatory responses to commensal bacteria, thereby maintaining tissue barrier functions [2]. Perturbation of TGF‐β signaling can promote inflammatory conditions such as Crohn's disease or ulcerative colitis. Mutations in TGF‐β pathway genes appear in 40–50% of colorectal cancers (CRCs) [3]. Common mutations affect the *SMAD4* gene (10–35%) and the *TGFBR2* gene (15% in microsatellite stable (MSS) and more than 90% in microsatellite instable (MSI) CRCs). TGF‐β also influences the tumor microenvironment by inducing gene expression changes in cancer‐associated fibroblasts (CAFs), thereby enhancing tumor aggressiveness, and by inducing immune suppression of tumor‐infiltrating cells of the innate and adaptive immune system [4, 5].

Interestingly, while TGF‐β acts as a tumor suppressor in normal colon epithelia and early‐stage colon cancer, it can induce epithelial‐to‐mesenchymal transition (EMT), which promotes tumor progression and metastasis, in advanced stages [6, 7]. EMT, a plastic and multistep process, is characterized by morphological changes of cells, facilitating their detachment and motility [8]. It was suggested that cancer cells retain some epithelial traits while they acquire mesenchymal characteristics, resulting in partial EMT states [9, 10, 11, 12]. The shift from tumor‐suppressive to tumor‐promoting functions is attributed to a decoupling of TGF‐β signaling from apoptotic pathways or the activation of non‐canonical pathways like PI3K‐AKT or Ras–ERK–MAPK [13, 14, 15, 16].

Advances in tumoroid models, which preserve molecular tumor characteristics, reflect tumor heterogeneity, and mimic patient response to therapies, have recently enhanced preclinical research [17]. These models were also used to study TGF‐β effects in physiologically relevant *in vitro* settings. For instance, murine‐derived colon tumoroids with mutations in *Apc*, *Kras*, and *p53* genes exhibited partial EMT and collective invasion upon TGF‐β treatment [18]. Conversely, human CRC patient‐derived tumoroids (PDTs) with active TGF‐β pathway status mostly displayed tumor‐suppressive effects, indicated by upregulation of CDK inhibitors, and in PDTs, which showed undisturbed proliferation, no signs of EMT were evident [4]. Thus, the effect of TGF‐β in CRC can be diverse and context‐dependent, and the mechanisms, which disconnect the tumor‐promoting effects from its tumor‐suppressive effects, are still not fully understood.

In this study, we employed a series of 10 human CRC PDTs from tumors of different locations, progression stage, and mutational backgrounds to explore the dual nature of TGF‐β effects in human CRC. PDTs with intact canonical TGF‐β signaling predominantly exhibited tumor‐suppressive and apoptotic responses to TGF‐β. In contrast, lines with mutations in TGF‐β pathway genes, or lines derived from CRC liver metastases, were less sensitive to the treatment. Notably, one PDT line derived from a primary tumor with *APC* and atypical *KRAS* ^Q22K^ mutation, but with intact TGF‐β signaling, displayed enhanced invasive properties, with phenotypic and molecular changes suggestive of partial EMT following TGF‐β1 exposure. In summary, these results highlight the importance of the mutational background of CRC cells to promote tumor progression via cancer cell‐intrinsic TGF‐β responses.

## 2 Materials and methods

### 2.1 PDT cultivation

PDT models were established as described in [19]. For cultivation, PDTs were maintained in droplets of Matrigel^®^ (Corning, Corning, NY, USA; Cat. No. 356231) in colon organoid medium as previously reported [20] without Wnt3a and R‐spondin, referred to as ENAS medium (Table 1) PDTs were routinely passaged at intervals of 7 days using TrypLE (Gibco, Schwerte, Germany; Cat. No. 12605010) and pipetting to dissociate the cells.

**Table 1:** Medium composition.

Component	Company, cat. No.	Final concentration
Basal medium		
Advanced DMEM/F12	Gibco, 12634‐010	
HEPES	Gibco, 15630056	10 mm
GlutaMAX™ Supplement	Gibco, 35050061	2 mm
Penicillin–Streptomycin (10 000 U/mL)	Gibco, 15140122	1×
Recombinant Human EGF	PeproTech, AF‐100‐15	50 ng/mL
ENAS medium = Basal medium + factors		
B‐27® Supplement (50X), serum free	Gibco,17504044	1×
N‐2 Supplement (100X)	Gibco, 17502001	1×
Nicotinamide	Sigma‐Aldrich, N0636	10 mm
N‐acetyl‐L‐cystein	Sigma‐Aldrich, A9165	1 mm
Recombinant Human Noggin	PeproTech, 120‐10C	100 ng/mL
A 83‐01	Sigma‐Aldrich SML0788	500 nm
SB 202190	Sigma‐Aldrich, S7076	10 μm
[Leu15]‐gastrin I, human	Sigma‐Aldrich, G9145	10 nm
ROCK inh. Y‐27632 2HCl	THP Medical Products, HY‐10583	10 μm

### 2.2 Mutational profile of PDTs

After genomic DNA was isolated with QIAamp DNA Mini Kit (Qiagen, Hilden, Germany; Cat. No. 51304), panel sequencing using the Ion AmpliSeq™ Cancer Hotspot Panel v2 (Thermo Fisher, Waltham, MA, USA; Cat. No. 4475346) was done to infer driver mutations of individual lines. In addition, whole exome sequencing was performed for PDT8 MSI line to identify mutations in *TGFBR2*.

### 2.3 Embedding of PDTs and immunohistochemistry

PDTs were fixed directly after culturing in 24‐well plates, with 4.5% paraformaldehyde (PFA). For fixation, Matrigel domes were mechanically disrupted by pipetting in 200 μL PFA, followed by 30 min incubation at room temperature (RT). The fixed PDTs were collected in a 1.5 mL tube and after centrifugation at 300 g/4 min/RT the pellet was washed with PBS and the centrifugation was repeated. To allow visualization of PDTs in subsequent steps, the PDT pellet was resuspended in 50 μL 1% Eosin, and incubated for 10 min at RT. After a washing step with PBS, the PDT pellet was resuspended carefully in 40 μL 0.8% agarose, which was prewarmed at 65 °C. PDTs in agarose were pipetted into a CultureWell™ reusable gasket (Grace Bio‐Labs, Bend, OR, USA; Cat. No. 103280). After drops were solidified for 30 min on ice, the agarose drops with PDTs were transferred into embedding cassettes. For additional fixation they were incubated for 15 min in PFA. Furthermore, the fixed PDTs in agarose were dehydrated and embedded into paraffin. After embedding in paraffin, 2 μm sections were prepared for hematoxylin and eosin (H&E) staining. Corresponding tumor tissues were used as 3 μm sections for H&E staining.

### 2.4 TGF‐β1 treatment of PDTs

Single cells were seeded at a concentration of 2000 cells/10 μL in 50% Matrigel/PBS domes. After 72 h in ENAS + ROCK inhibitor, medium was replaced to different conditions: ENAS + solvent (0.2 mm HCL/PBS + bovine serum albumin (BSA)), ENAS + 5 ng·mL^−1^ TGF‐β1 (Recombinant Human TGF‐β1 Protein, R&D Systems, Minneapolis, MN, USA; Cat. No. 240‐B), basal medium + solvent, basal medium + 5 ng·mL^−1^ TGF‐β1. PDTs were treated for a time course of 10 days and medium was changed every other day. Detailed medium composition is provided in Table 1.

### 2.5 KRAS inhibitor treatment of PDTs and combination treatment with TGF‐β1

For dose response curves, single cells were seeded at a concentration of 2000 cells/8 μL in 50% Matrigel/PBS domes. After 72 h in ENAS + ROCK inhibitor, medium was replaced to ENAS + respective KRAS inhibitors. For BI‐2865 (MedChemExpress, Monmouth Junction, NJ, USA; # HY‐153724) treatment a concentration range from 0.014 to 50 μm was used and viability was measured after 72 h of treatment. For ACBI3 (Boehringer Ingelheim, Ingelheim, Germany; opnMe^®^) a concentration range from 0.0046 to 30 μm was used and viability was measured after 6 days of treatment as described below (2.6). For the combination treatment with TGF‐β1 single cells were seeded at a concentration of 2000 cells/10 μL in 50% Matrigel/PBS domes. After 72 h in ENAS + ROCK inhibitor, medium was replaced to basal medium + solvent or basal medium + 5 ng·mL^−1^ TGF‐β1 with or without KRAS inhibitors (BI‐2865: 0.37 μm; ACBI3: 0.12 μm). Viability was measured after 10 days of treatment.

### 2.6 Viability assays

Single cells were seeded at a concentration of 2000 cells/10 μL, as 8 μL 50% Matrigel/PBS domes in white 96‐well plates as technical triplicates. Treatment was carried out as stated in 2.4 and 2.5. CellTiter‐Glo 3D Cell Viability Assay (Promega, Madison, WI, USA; Cat. No. G9681) was used to detect viability. For this, medium was removed and 75 μL fresh basal medium without EGF and 75 μL CellTiter‐Glo 3D reagent were added. Measurement was carried out according to manufacturer's instructions using plate reader synergy H1 (Bio Tek Agilent, Santa Clara, CA, USA). The following formula was used to calculate well viability: well viability = ((well value − average positive control)/(average vehicle control − average positive control)) * 100, whereby positive control refers to Staurosporin 5 μm control (Sigma‐Aldrich, St. Louis, MO, USA; Cat. No. 569397), and the vehicle control refers to ENAS + solvent. Values were plotted in graphpad prism version 8 (GraphPad, Boston, MA, USA).

### 2.7 Immunofluorescence staining

PDT single cells were prepared and seeded into 8‐well glass bottom slides (IBIDI, Gräfelfing, Germany; Cat. No. 80827) at a density of 1000 cells/15 μL within 50% Matrigel/PBS domes. Following a 10‐day TGF‐β1 treatment, PDTs underwent fixation with 1% PFA for 20 min at RT, followed by a secondary fixation (1% PFA, PBS, 0.1% Triton‐X 100) for another 20 min. Fixation was carried out under constant mild shaking (40–50 rpm) at RT as all following steps, if not otherwise stated. After fixation, wells were rinsed three times with PBS. Permeabilization involved a 30‐min incubation in 200 μL PBS solution with 0.1% Triton‐X 100, followed by three washes with washing buffer (PBS, 0.1% Triton‐X 100, and 0.05% Tween). For blocking, a mixture of PBS, 0.1% Triton‐X 100, and 10% goat serum was used, for 1 h. The primary antibody (Table 2), diluted in blocking solution, was added (150 μL per well) and incubated overnight with gentle shaking in a humidified chamber at 4 °C. The next day entailed three washes for 10 min each with washing buffer, followed by a 2‐h incubation with 50 μL of secondary antibody (Table 2), in blocking solution in darkness at RT. After three 15‐min washes in washing buffer, wells were incubated with 150 μL DAPI (1 : 50 000) for 20 min, followed by a final wash and storage in 200 μL PBS at 4 °C in the dark. Imaging was performed using a Zeiss Axiovert 200M (Zeiss, Oberkochen, Germany) for light microscopy and a Zeiss LSM 700 for confocal immunofluorescence.

**Table 2:** List of antibodies for immunofluorescence (IF) and western blotting.

Antigen	Supplier, cat. number	Host species, dilution
Primary antibodies for IF		
Fibronectin	Cell Signaling, #26836	Rabbit, 1/400–1/800
E‐Cadherin	Cell Signaling, #3195	Rabbit, 1/250
Ki67	Cell Signaling, #9129	Mouse, 1/400–1/500
Slug	Cell Signaling, #9585	Rabbit, 1/200
PAX6	Cell Signaling, # 60433	Rabbit, 1/200
Primary antibodies for western blot		
Pospho‐SMAD2	Cell Signaling, #3108	Rabbit, 1/500 in BSA
Pospho‐SMAD3	Abcam, #ab52903	Rabbit, 1/2000 in BSA
SMAD2/3	Cell Signaling, #8685	Rabbit, 1/1000 in BSA
Phospho‐Erk1/2	Cell Signaling, #4370	Rabbit, 1/1000 in BSA
ERK	Cell Signaling, #4695	Rabbit, 1/1000 in Milk
BIM	Cell Signaling, #2933	Rabbit, 1/1000 in BSA
Bcl‐xL	Cell Signaling, #2764	Rabbit, 1/1000 in BSA
E‐Cadherin	Cell Signaling, #3195 T	Rabbit, 1/1000 in BSA
Keratin 20	Cell Signaling, #13063	Rabbit, 1/1000 in BSA
Fibronectin	Cell Signaling, #26836S	Rabbit, 1/500 in BSA
Fascin	Cell Signaling, #9269	Rabbit, 1/1000 in BSA
Slug	Cell Signaling, #9585	Rabbit, 1/1000 in BSA
LGR5	Sigma, #HPA012530	Rabbit, 1/1000 in BSA
ANPEP	Cell Signaling, #32720	Rabbit, 1/1000 in BSA
CEACAM1	Cell Signaling, #5441	Rabbit, 1/1000 in BSA
PAX6	Cell Signaling, #60433	Rabbit, 1/1000 in BSA
β‐tubulin	Cell Signaling, #86298	Mouse, 1/3000 in BSA
β‐Actin	Proteintech, #66009‐1‐lg	Mouse, 1/2000 in BSA

Name (species reactivity/ host)	Conjugate	Supplier, cat. number, dilution
Secondary antibodies IF		
Goat anti‐Rabbit IgG (H + L) Highly Cross‐Adsorbed Secondary Antibody	Alexa Fluor™ 488	Invitrogen, Catalog # A‐11034, 1/500
F(ab′)2‐Goat anti‐Rabbit IgG (H + L)	Alexa Fluor™ 488	Invitrogen, Catalog # A‐11070, 1/500
F(ab′)2‐Goat anti‐Mouse IgG (H + L)	Alexa Fluor™ 546	Invitrogen, Catalog # A‐11018, 1/500
Secondary antibodies for western blot		
Peroxidase AffiniPure™ Rabbit Anti‐Mouse IgG, Fcγ fragment specific	Horseradish Peroxidase	JacksonimmunoResearch Catalog: 315–035‐008
Peroxidase AffiniPure™ F(ab′)₂ Fragment Goat Anti‐Rabbit IgG, F(ab′)₂ fragment specific	Horseradish Peroxidase	JacksonimmunoResearch Catalog: 111‐036‐047

### 2.8 Protein isolation and western blotting

To collect PDTs, the culture medium was removed, the domes were rinsed with PBS and resuspended in 350 μL Cell Recovery Solution (Corning™, Cat. No. CLS354253). The mixture was incubated for 45 min at 4 °C, then washed with cold PBS. PDTs were harvested by centrifugation. Post‐collection, the pellets were snap‐frozen and subsequently stored at −80 °C. Protein isolation and western blotting was carried out as described earlier [21], with minor deviations: 10 μg protein was used for each blot and BSA was used for blocking of membranes. Antibodies used are provided in Table 2.

### 2.9 Trans‐well invasion assay

35 000 single cells were seeded in 50 μL 10% Matrigel/PBS into Matrigel coated trans‐well plates (Corning, Cat. No. 354480). After 72 h in ENAS + ROCK inhibitor, PDTs were cultured for 5 days in basal medium + solvent or 5 ng·mL^−1^ TGF‐β1. In lower wells basal medium was added. After this pre‐treatment, PDTs were treated further in these conditions, and in the lower well, 300 μL basal medium + 10% fetal calf serum (FCS) as chemoattractant was added. As negative control (no attraction) basal medium only was added. After 3 days, medium (+/− TGF‐β1) was changed in upper wells, and in lower wells 300 μL of medium with or without FCS was added. After 5 days of chemoattraction, cells, which migrated through the membrane and were attached at the lower side of the mesh, were fixed with 100% methanol, and stained with crystal violet (Sigma‐Aldrich, Cat. No. C6158). Quantification of the covered area was performed using Fiji software [22].

### 2.10 RNA isolation and gene expression analysis

RNA was isolated directly after treatment with RNeasy Kit (Qiagen, Cat. No. 74104) using the on‐column DNA digestion protocol following the manufacturer's instructions and further processed for RNA sequencing or quantitative reverse‐transcription PCR (qRT‐PCR). For qRT‐PCR, iScript™ cDNA Synthesis Kit (Bio Rad, Hercules, CA, USA; Cat. No. 1708891) was used for cDNA preparation and Luna^®^ Universal qPCR Master Mix (NEB, Ipswich, MA, USA; Cat. No. M3003L) was used for subsequent qPCR. Relative gene expression levels were calculated with the delta CT method using TATA‐Box Binding Protein (*TBP*) as a reference gene (Primers are listed in Table 3). Each sample was measured in technical triplicates.

**Table 3:** List of primers for qRT‐PCR. All sequences are listed in 5′‐3′ direction.

Gene	Fwd	Rev
*ANPEP*	ATGCTTCCCAAAGGTCCCAG	ACTGACAATGAAGGCCAGCA
*SPON2*	TGGTCTCGTTTGTGGTGC	GGAGGACGTTATCTCGGTCA
*CEACAM1*	AAGCGACCAGCGTGATCT	AAAGTTCAGGGTAGAATAAGTAACTTCA
*TFF3*	CTTGCTGTCCTCCAGCTCT	CCGGTTGTTGCACTCCTT
*MUC2*	TCCTCCCAGCAGAACAACAC	CGAAGTGCTCCCCAAACTCT
*LGR5*	CACGTACCCACAGAAGCTCT	TTTTGTTCAGGGCCAAGGTC
*STNM1*	ATTCCCCCTTTCCCCTCCAA	CCAGCTGCTTCAAGACCTCA
*PAX6*	GCAACCTGGCTAGCGAAAAG	TTCTCTCCCCCTCCTTCCTG
*LYZ*	TTTCTGTTACGGTCCAGGGC	CAGTGCTTCTGTCTCCAGCA
*CHD1*	TGCACCAACCCTCATGAGTG	GTCAGTATCAGCCGCTTTCAG
*KRT20*	GGTCGCGACTACAGTGCATA	TCCTCAGCAGCCAGTTTAGC
*KRT8*	ACCCAGGAGAAGGAGCAGAT	ATGTTGCTTCGAGCCGTCTT
*FN1*	TCCCTCGGAACATCAGAAAC	CAGTGGGAGACCTCGAGAAG
*VIM*	TTGCAGGAGGAGATGCTTCA	TTCGTGGAGTTTCTTCAAAAAGG
*AXL*	TCAAGGTGGCTGTGAAGACGATGA	AACCCTGGAAACAGACACCGATGA
*FSCN*	ATCAACCGCCCCATCATCGT	CGAAGAAGAAGTCCACAGGAGT
*SNAI2*	GCCTCCAAAAAGCCAAACTACA	GAGGATCTCTGGTTGTGGTATGACA
*L1CAM*	TGGCAGGAGCAGATTGTCAG	TCAATGCCTTCCAGCTCAGG
*MMP14*	TTGTCTCCTGCTCCCCCTG	GTGTGTGGGTACGTAGGTCC
*ITGA5*	GTTGCATTTCCGAGTCTGGG	TTCGGTAGGGCATCTTCAGG
*TBP*	TTCGGAGAGTTCTGGGATTG	TTCGTGGCTCTCTTATCCTCA

#### 2.10.1 RNA sequencing and bioinformatics analysis

Isolated RNA was processed for RNA sequencing by the Biomedical Sequencing Facility at the Research Center for Molecular Medicine (CeMM) of the Austrian Academy of Sciences. Total RNA was quantified via Qubit 2.0 (Thermo Fisher) and integrity was assessed with Bioanalyzer 2100 (Agilent, Santa Clara, CA, USA). RNA sequencing libraries were prepared using NEBNext^®^ Ultra™ II DNA Library Prep Kit for Illumina^®^ (San Diego, CA, USA; Cat. No. E7645S) and quantified as DNA via Qubit 2.0. Library size distribution was evaluated with Bioanalyzer 2100 (Agilent). Samples were equimolarly pooled for next‐generation sequencing. Libraries for expression profiling were sequenced using HiSeq 3000 (Illumina) with a 50‐base‐pair single‐end protocol.

##### 2.10.1.1 Pre‐processing

FASTQ paired‐end reads were extracted from BAM files provided by the sequencing facility using bamToFastq from bedtools [23], while cutadapt [24] was used to remove unwanted sequences (e.g., adapters, poly‐A tails, etc.) and low‐quality reads. FastQC [25] and MultiQC [26] were both used to check the quality of reads and evaluate potential outliers. Then, the STAR aligner tool [27] was used to map the reads to the GRCh38.p13 (release 107) human reference genome, obtained from ENSEMBL (https://www.ensembl.org/Homo_sapiens/Info/Index), and then count mapped reads to each gene. A total of 61 860 transcripts were successfully mapped to the genome. A naive pre‐filtering step was performed to remove low count genes by only keeping those transcripts with an average count across all samples bigger than one. This yielded a total of 25 296 transcripts for further analysis.

##### 2.10.1.2 Differential expression analysis

The R/Bioconductor package DESeq2 [28] was used to perform differential gene expression (DE) analysis of the un‐stranded STAR transcript counts. The read counts were normalized by the DESeq2 normalization method of variance stabilizing transformation (VST), to be used for comparative analysis. Differentially expressed genes (DEGs) were derived from likelihood ratio tests in the respective treatment condition from ET versus ES, BS versus ES, BT versus ES, BS versus ET, BT versus ET and BT versus BS. Genes with an adjusted *P*‐value (*P*adj) < 0.05 and absolute log_2_ fold change (LFC) > 1 were considered significantly differentially expressed, representing a conservative and stringent approach. Figures from this analysis, such as PCA plots and heatmaps, were generated using R/Bioconductor packages ggplot2 [29] and Complexheatmap [30].

##### 2.10.1.3 Functional gene set enrichment analysis (GSEA) for DEGs

To gain insight into the biological relevance of the significantly DEGs in each experimental subgroup, enrichment analysis was performed using the R/Bioconductor package clusterProfiler [31]. Enriched pathways were mined using Reactome [32] database.

##### 2.10.1.4 Marker gene expression for colon crypt‐specific cell types

Marker genes for different colon cell types were inferred from single cell RNA sequencing data available from PangloaDB [33]. Gene expression of marker genes was analyzed for the different conditions (ES, BS, BT) and plotted as VST gene value counts using Complexheatmap [30]. Row means were subtracted for better visualization of differences.

##### 2.10.1.5 Consensus molecular subtypes (CMS) calling

We applied the cmscaller r Package [34] to raw gene counts to detect CMS subtypes of PDTs following different treatments.

##### 2.10.1.6 Expression of EMT signature genes

EMT signature genes were obtained from [35] and assessed for their differential expression in BS vs ES and BT vs BS. Significantly DEGs (*P*adj < 0.05 and absolute LFC > 1) between the indicated conditions were plotted using the R software's ggplot2 [29] package.

##### 2.10.1.7 Gene set enrichment analysis (GSEA)

Gene overlap and GSEA were done using the GSEA website (https://www.gsea‐msigdb.org/gsea/msigdb/human/annotate.jsp) and GSEA software version 4.3.3 (Mac App) [36, 37]. The gene list from the Hallmark EMT signature of the Molecular Signatures Database (MSigDB) was used for GSEA [38]. Data visualization, including bubble charts, was done using ggplot2 [29]. All analyses were conducted using r version 4.3.1.

### 2.11 Statistics

Statistical evaluations were conducted using prism software (GraphPad) version 8. The analysis incorporated a one‐way analysis of variance (ANOVA), followed by Tukey's multiple comparison test or Welch's *t*‐test, with 95% confidence interval and significance was determined at *P*adj < 0.05.

### 2.12 Ethics declaration

All experiments were performed adhering to the “Guidelines for Good Scientific Practice” and in alignment with the most recent “Declaration of Helsinki.” Patient material was collected at the General Hospital of Vienna (AKH) between June 2017 and July 2023 and used upon written informed consent, only. The ethics governing this research received approval from the IRB of the Medical University of Vienna (N1248/2015).

## 3 Results

### 3.1 Generation of PDTs encompassing a spectrum of CRC progression

**Fig. 1:**
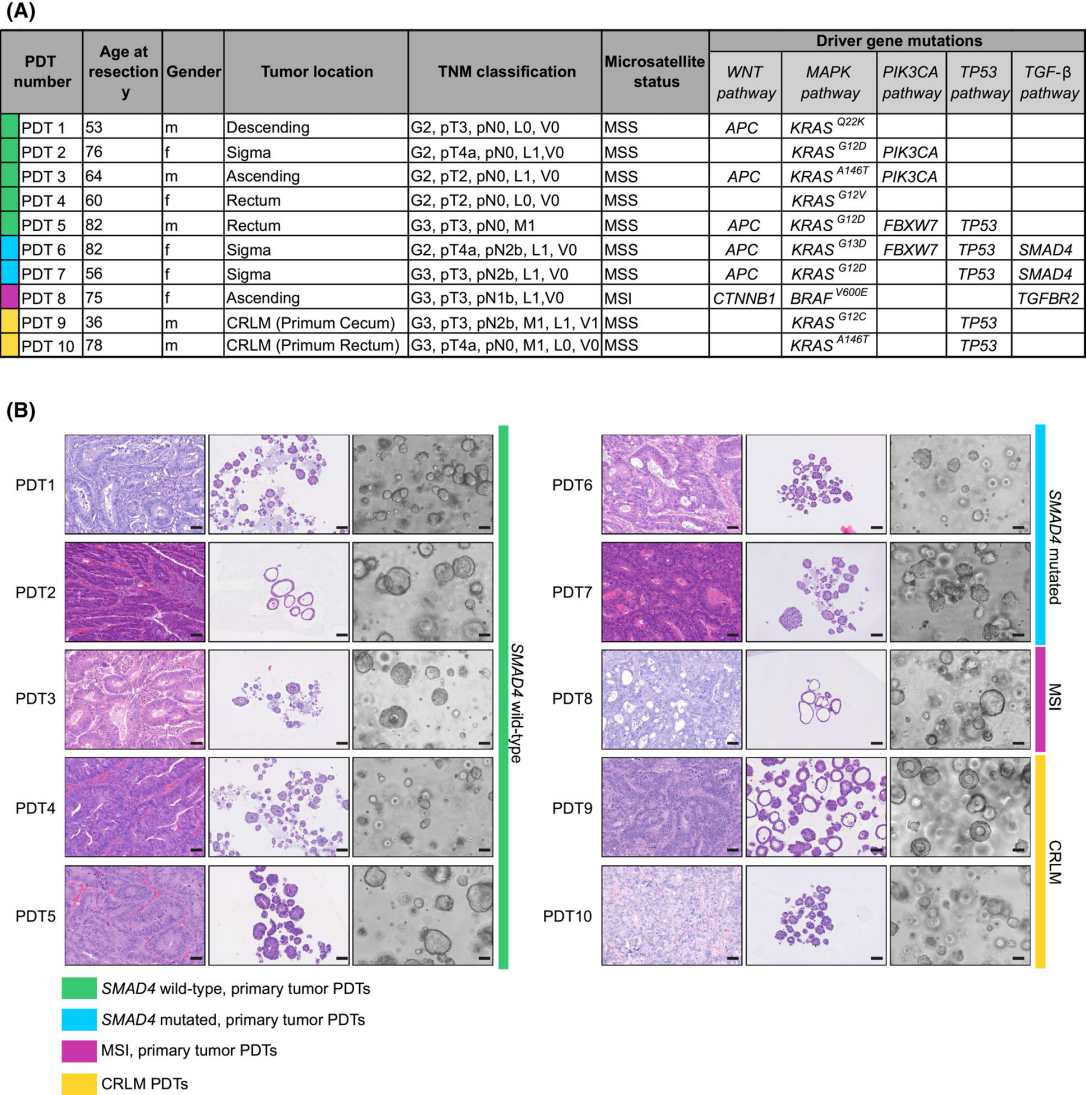
Molecular and phenotypic characteristics of colorectal cancer (CRC) patient‐derived tumoroid (PDT) lines. (A) Demographic table of different PDT lines used in this study, including patient age in years (y), gender (m: male; f: female), tumor location, TNM classification, microsatellite status (MSI, microsatellite instable; MSS, microsatellite stable), and driver gene mutations. The colors in the first row of the table group PDTs in four different classes. Green: *SMAD4* wild‐type, primary tumor PDTs; blue: *SMAD4* mutated, primary tumor PDTs; purple: MSI, primary tumor PDTs; yellow: colorectal liver metastasis (CRLM) PDTs. (B) Histo‐morphological analysis of PDT lines compared to their tumors of origin. Hematoxylin and eosin (H&E) staining of formalin‐fixed paraffin‐embedded (FFPE) sections of original tumor tissues (left) compared to PDT lines cultured for 7 days in ENAS medium (middle). Bright‐field microscopic images of PDT lines cultivated for 7 days in ENAS medium (right). Scale bars: 50 μm. PDTs in B are grouped, and color coded as described in A.

TGF‐β elicits distinct responses in cancer cells depending on their state of progression and other contributing factors [39]. Our study aimed to investigate this variability of TGF‐β response in CRC PDTs. We utilized 10 different PDT lines derived from tumors with varying cancer grades (G2 or G3), and originating from different tumor locations, thereby covering a spectrum of CRC progression (Fig. 1A). Nine of the lines were *KRAS* mutant, while one MSI line harbored a *BRAF* mutation. During disease progression, CRC cells may develop resistance to TGF‐β by acquiring mutations in key players of the canonical TGF‐β signaling pathway, such as TGF‐β receptors or SMAD proteins [3]. Consequently, we grouped the PDTs generated from primary tumors into *SMAD4* wild‐type (PDT1‐PDT5) and *SMAD4* mutated (PDT6 and PDT7) lines. Moreover, the included MSI line carried a frameshift mutation in *TGFBR2* (PDT8), while two lines isolated from liver metastatic lesions had no mutations in TGF‐β pathway genes (PDT9, PDT10). The included PDT lines did not only differ in their mutational background and origin but also exhibited morphological diversity and predominantly mirrored the morphology of the original tumor tissue (Fig. 1B).

### 3.2 TGF‐β1 treatment elicits divergent responses in CRC PDTs

**Fig. 2:**
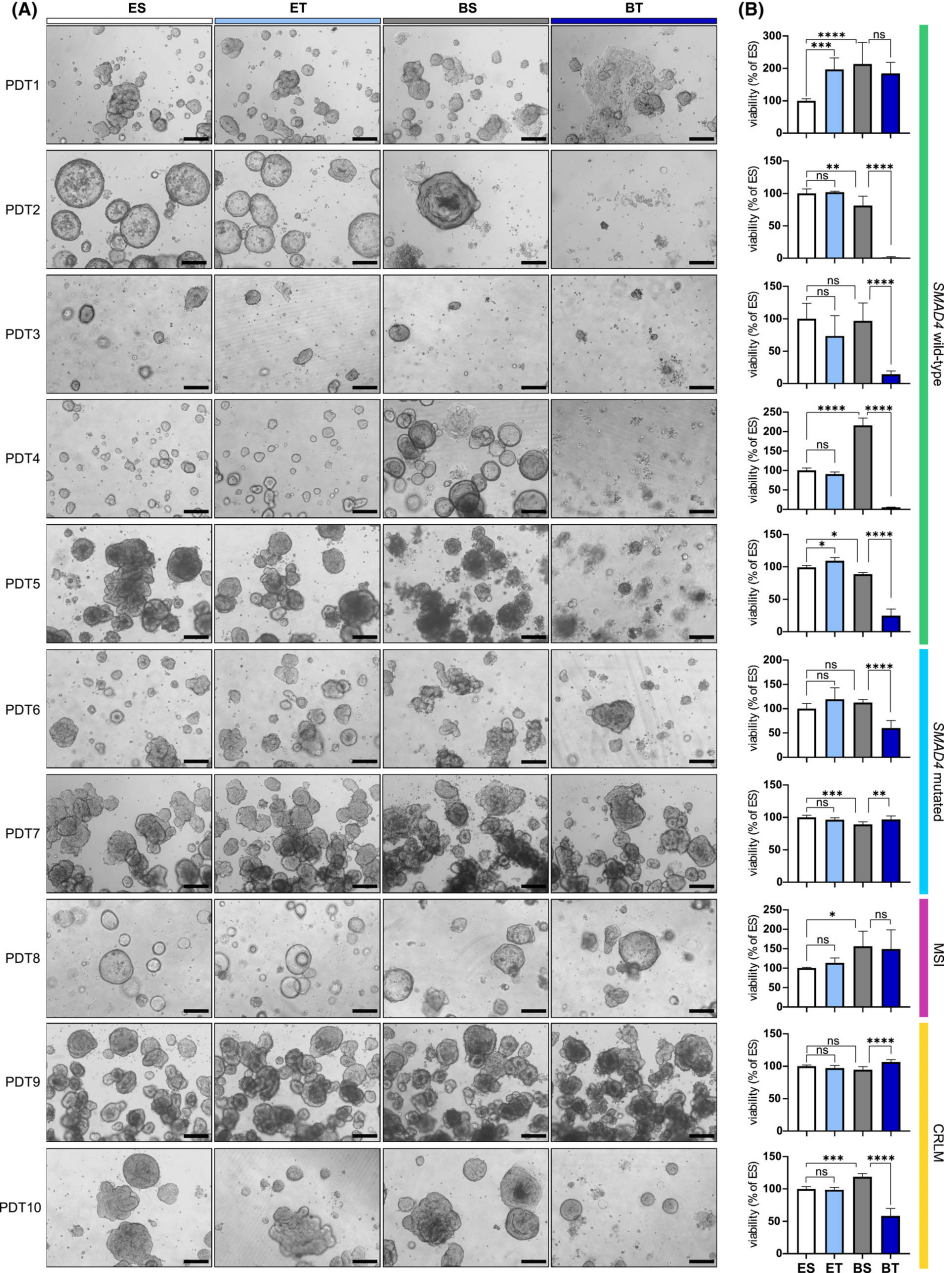
TGF‐β1 treatment induces different responses in individual patient‐derived tumoroids (PDTs). (A) Representative bright‐field microscopic images of different PDT lines cultured in different media conditions for 10 days, ES: ENAS + solvent (white); ET: ENAS + 5 ng·mL^−1^ TGF‐β1 (light blue), BS: basal medium + solvent (gray); BT: basal medium + 5 ng·mL^−1^ TGF‐β1 (dark blue) (*n* = 2). Scale bar: 200 μm. PDTs are grouped into four different classes indicated at the right in different colors. Green: *SMAD4* wild‐type, primary tumor PDTs; blue: *SMAD4* mutated, primary tumor PDTs; purple: microsatellite instable (MSI), primary tumor PDTs; yellow: colorectal liver metastasis (CRLM) PDTs. (B) Cell viability of PDTs in different media conditions as in (A) measured with CellTiter‐Glo® 3D Cell Viability Assay. Viability is presented as % of viability relative to ES. Graphs show mean and standard deviation (SD) from three technical replicates of two individual experiments (*n* = 2). Statistical significance was calculated with graphpad prism version 8 using ordinary one‐way ANOVA followed by Tukey's multiple comparison test with 95% confidence interval: ns *P* > 0.05; *P ≤ 0.05; **P ≤ 0.01; ***P ≤ 0.001; ****P ≤ 0.0001.

To study the responses of the different lines to TGF‐β1, the cell culture medium was adapted from standard organoid medium (ENAS, Table 1), which had been developed for the growth of stem cells and contains TGF‐β inhibitors among several other factors, to basal medium containing EGF as the only growth factor. After 10 days of culturing, significant differences in the response of PDTs to the medium composition and TGF‐β1 treatment were observed (Fig. 2A,B). Some *SMAD4* wild‐type lines including PDT2, 4 and 8 showed morphological changes upon culture in basal medium. For instance, PDT2 exhibited substantial morphological changes, transitioning from large, single‐layered cystic PDTs to denser structures with thickened epithelium, indicating differentiation of PDTs [40]. As expected, long‐term TGF‐β1 treatment for 10 days had no suppressive effects on PDTs cultured in ENAS medium, due to the presence of TGF‐β inhibitors in the medium. However, a tumor‐suppressive effect of TGF‐β1 was evident in most of the primary *SMAD4* wild‐type PDTs in basal medium (PDT2‐5), while lines carrying mutations in the TGF‐β signaling pathway were less sensitive to TGF‐β1 treatment (PDT6,7,8). Notably, PDTs derived from metastatic tumors (PDT9,10) showed only minor or no reduction in viability following TGF‐β1 treatment in basal medium. Interestingly, the viability of PDT1, which was derived from a primary tumor with intact TGF‐β signaling, was not affected by TGF‐β1 administration. Upon treatment, the morphological changes, which were apparent in basal medium, were further enhanced in this line exhibiting extended 2D growth patterns (27%, Fig. S1).

**Fig. 3:**
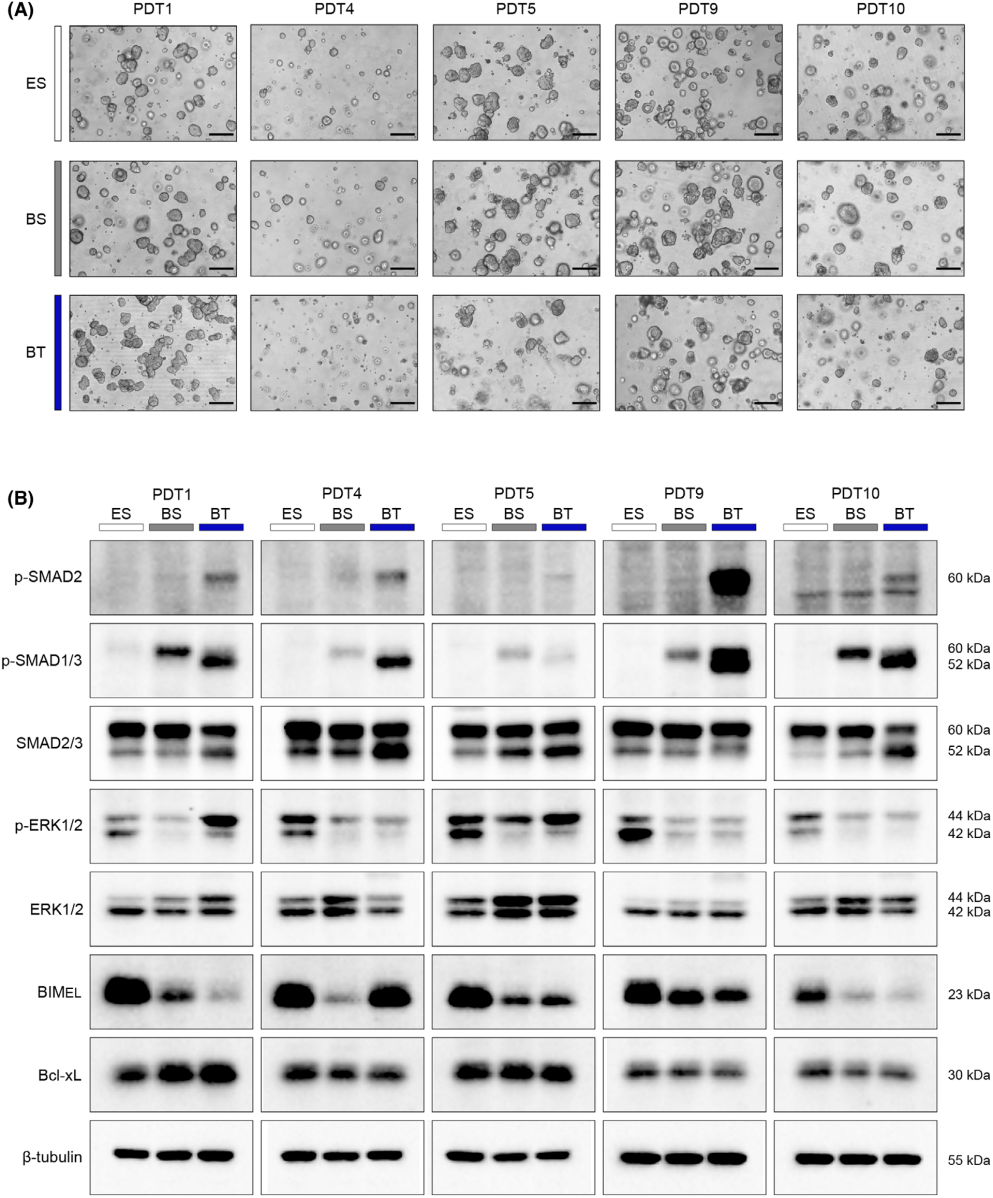
TGF‐β1 induces different signaling pathways in insensitive and sensitive patient‐derived tumoroids (PDTs). (A) Representative bright‐field microscopic images of *SMAD4* wild‐type PDT lines (PDT1,4,5: primary PDTs; PDT9,10: colorectal liver metastasis (CRLM) PDTs) cultured in different conditions for 5 days (*n* = 2). ES: ENAS + solvent; BS: basal medium + solvent; BT: basal medium + 5 ng·mL^−1^ TGF‐β1. Scale bar: 200 μm. (B) Western blot analysis of PDTs treated as in (A) for phospho‐SMAD2 (p‐SMAD2) (60 KDa), phospho‐SMAD1/3 (p‐SMAD1/3) (60 kDa, 52 kDa), total SMAD2/3 (60 kDa, 52 kDa), phospho‐ERK1/2 (p‐ERK1/2) (44 kDa, 42 kDa), total ERK1/2 (44 kDa, 42 kDa), BIM_EL_ (23 kDa), Bcl‐xL (30 kDa) and β‐tubulin (55 kDa) as loading control (*n* = 2).

In order to investigate the different effects of TGF‐β1 treatment in primary and metastatic *SMAD4* wild‐type lines in more detail, we assessed their phenotypic and molecular changes in ENAS medium compared to basal medium +/− TGF‐β1 for 5 days. Within this time period, the growth‐suppressive effects of TGF‐β1 were less pronounced for PDT4, 5 and 9, allowing for the analysis of TFG‐β signaling on protein level, while PDT2 and 3 showed high sensitivity and could not be recovered after 48 h of treatment (Fig. 3A and data not shown). Similar to 10‐day treatment, PDT1 and PDT9 were unaffected by TGF‐β1 treatment, although no morphological changes and 2D growth patterns were observed for PDT1 upon short term treatment. All lines except for PDT5 exhibited high levels of SMAD2 and/or SMAD3 signaling, reflected by SMAD2/3 phosphorylation in PDTs cultivated in basal media containing TGF‐β1, and upregulation of total SMAD3 protein for most of the lines (Fig. 3B). Moreover, phosphorylated SMAD1 protein was detectable in basal medium, most likely due to activated BMP signaling resulting from the lack of Noggin in this condition. The effects of TGF‐β1 were well reflected in the protein levels of the pro‐apoptotic factor BIM [13, 41], which was strongly downregulated in PDT1 and expressed at low levels in PDT5, 9 and 10. Conversely, PDT4 exhibited elevated BIM protein levels, suggesting increased pro‐apoptotic signaling, elucidating the tumor‐suppressive effects of the treatment. Furthermore, BIM protein levels were anti‐correlated to phospho‐ERK levels following TGF‐β1 treatment in PDT1 and PDT5. Activated ERK was previously shown to promote BIM phosphorylation resulting in its degradation [42]. ERK activation was not detected in the metastatic lines, suggesting different signaling pathways promoting BIM downregulation in advanced stages. Furthermore, the expression of the pro‐survival factor Bcl‐xL was induced in the PDT1 line upon TGF‐β1 administration, additionally reinforcing the survival advantage of this line compared to the other lines.

To test whether the insensitivity of PDT1 towards TGF‐β1 was dependent on KRAS signaling, we used the panKRAS inhibitor BI‐2865 [43] and the KRAS degrader ACBI3 [44], which both showed high efficacy against PDT1 (Fig. S2A,B). Notably, co‐treatment of either drug with TGF‐β1 for 10 days resulted in significantly reduced viability compared to KRAS inhibitor only treatment (Fig. S2C,D).

Together, these data indicate that TGF‐β1 responses of PDTs largely reflect the diverse *in vivo* effects of TGF‐β signaling. While tumor‐suppressive consequences were observed in most of the primary *SMAD4* wild‐type PDTs, metastatic lines were less sensitive, and the presence of an atypical KRAS^Q22K^ mutation rendered PDT1 insensitive to the inhibitory effects of TGF‐β1.

### 3.3 PDT culture in basal medium induces differentiation towards specialized cell types of the colon

**Fig. 4:**
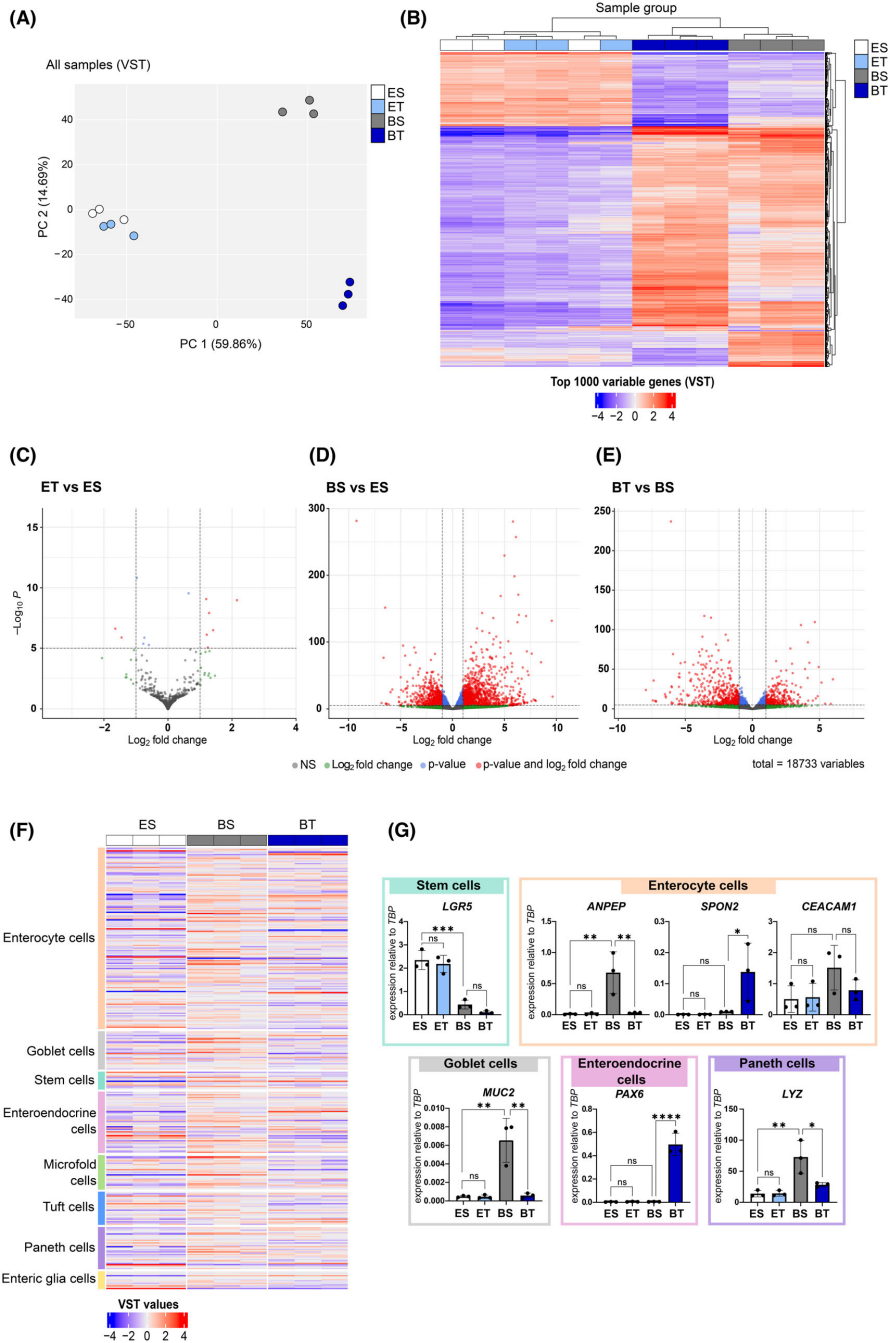
Gene expression analysis reveals differentiation of patient‐derived tumoroids (PDTs) to specific cell types of the colon crypt in basal medium. (A) Principal component analysis (PCA) of RNA sequencing data of PDT1 cultured in different conditions based on variance stabilizing transformation (VST) read counts (*n* = 3). Colors indicate different conditions: white = ES: ENAS + solvent; light blue = ET: ENAS + 5 ng·mL^−1^ TGF‐β1; gray = BS: basal medium + solvent; dark blue = BT: basal medium + 5 ng·mL^−1^ TGF‐β1. (B) Dendrogram and heatmap showing unsupervised hierarchical clustering of the top 1000 variable expressed genes using normalized read counts (VST transformed counts in DESeq2) of PDT1 cultured as in (A) (*n* = 3). Columns represent individual samples (color coded as in A), rows represent individual genes. The color gradient on the bottom shows VST normalized counts, with blue indicating below‐average gene expression and red indicating above‐average expression. (C–E) Volcano plots of significant differentially expressed genes between different media conditions as in (A) (*n* = 3). Significantly deregulated genes are indicated in red with *P*adj < 0.05 and Log_2_ fold change (LFC) > 1 or < −1. Genes below this significance threshold are indicated in gray (*P*adj > 0.05. LFC < 1 and > −1), green (*P*adj > 0.05. LFC > 1 or < −1), and blue (*P*adj < 0.05. LFC < 1 and > −1). (F) Heatmap showing VST gene count values of PDT1 in different conditions (ES, BS, BT) for colon crypt cell type‐associated genes inferred from [33] (https://panglaodb.se/) (*n* = 3). Cell types are ordered according to their frequency in the colon crypt. Red indicates upregulation, blue downregulation. (G) Gene expression analysis of indicated cell type‐associated genes in different conditions as in (A) quantified by qRT‐PCR. Data are represented as expression relative to TATA‐Box Binding Protein (*TBP*) as housekeeping gene. Bar graphs show mean and standard deviation (SD) of three independent experiments, whereby each dot represents the mean of three technical replicates (*n* = 3). Statistical significance was calculated using graphpad prism version 8 with ordinary one‐way ANOVA followed by Tukey's multiple comparison test with 95% confidence interval: ns *P* > 0.05; *P ≤ 0.05; **P ≤ 0.01; ***P ≤ 0.001; ****P ≤ 0.0001.

To further investigate the effects of TGF‐β1 treatment on the primary PDT1 line, which showed no change in viability but strong morphological differences, we assessed the gene expression changes of this specific line under different medium conditions and following TGF‐β1 treatment. Principal component analysis (PCA) revealed clustering of PDTs cultivated in ENAS +/− TGF‐β1, whereas PDTs cultivated in basal medium with or without TGF‐β1 showed additional significant alterations and clustered separately (Fig. 4A). Unsupervised hierarchical clustering based on the top 1000 most variable expressed genes grouped PDTs cultured in ENAS medium apart from PDTs grown in basal medium +/− TGF‐β1 (Fig. 4B). Similarly, differential gene expression analysis resulted in only 28 significantly deregulated genes (18 up, 10 down) (*P*adj < 0.05. absolute LFC > 1) in ENAS versus ENAS‐TGF‐β1 treated PDTs (Fig. 4C, Table S1), while 3737 significantly deregulated genes (2197 up, 1540 down) were detected in the ENAS versus basal medium comparison (Fig. 4D, Table S1). Moreover, a strong impact of TGF‐β1 treatment was observed in basal medium, with 1570 significantly deregulated genes (647 up, 923 down) (Fig. 4E, Table S1).

Next, we explored gene expression levels of marker genes, which are characteristic for various cell types of the colon crypts [33]. Notably, genes associated with enterocytes and secretory cells were generally upregulated in basal medium conditions compared to ENAS, whereas stem cell markers were downregulated (Fig. 4F, Fig. S3.1, Table S2). Upon addition of TGF‐β1, the expression of most of these cell type‐associated genes was dampened. Interestingly, some specific genes such as *SPON2*, a gene associated with enterocytes and CRC progression [45], *PAX6*, encoding a TF associated with enteroendocrine differentiation [46], and the goblet cell‐associated gene *KRT7*, which has been linked to metastasis [47], were specifically upregulated in basal medium with TGF‐β1 (Fig. S3.1, Table S2). The expression of several genes was confirmed by qRT‐PCR also highlighting the downregulation of the stem cell marker *LGR5* in basal medium conditions, and altered expression of enterocyte (*ANPEP*, *SPON2*, *CEACAM1*), goblet (*MUC2*), enteroendocrine (*PAX6*) and Paneth cell (*LYZ*) associated genes in the different conditions (Fig. 4G). Moreover, downregulation of LGR5 and upregulation of ANPEP, CEACAM1, and PAX6 was further confirmed on protein level (Fig. S3.2).

Taken together, these data demonstrate the upregulation of genes associated with different colon crypt cell populations upon withdrawal of stem cell niche factors and an additional deregulation of genes involved in tumor progression and metastasis upon TGF‐β1 stimulation.

### 3.4 TGF‐β1 treatment of PDT1 in basal medium shifts the differentiation of PDTs towards a mesenchymal phenotype

**Fig. 5:**
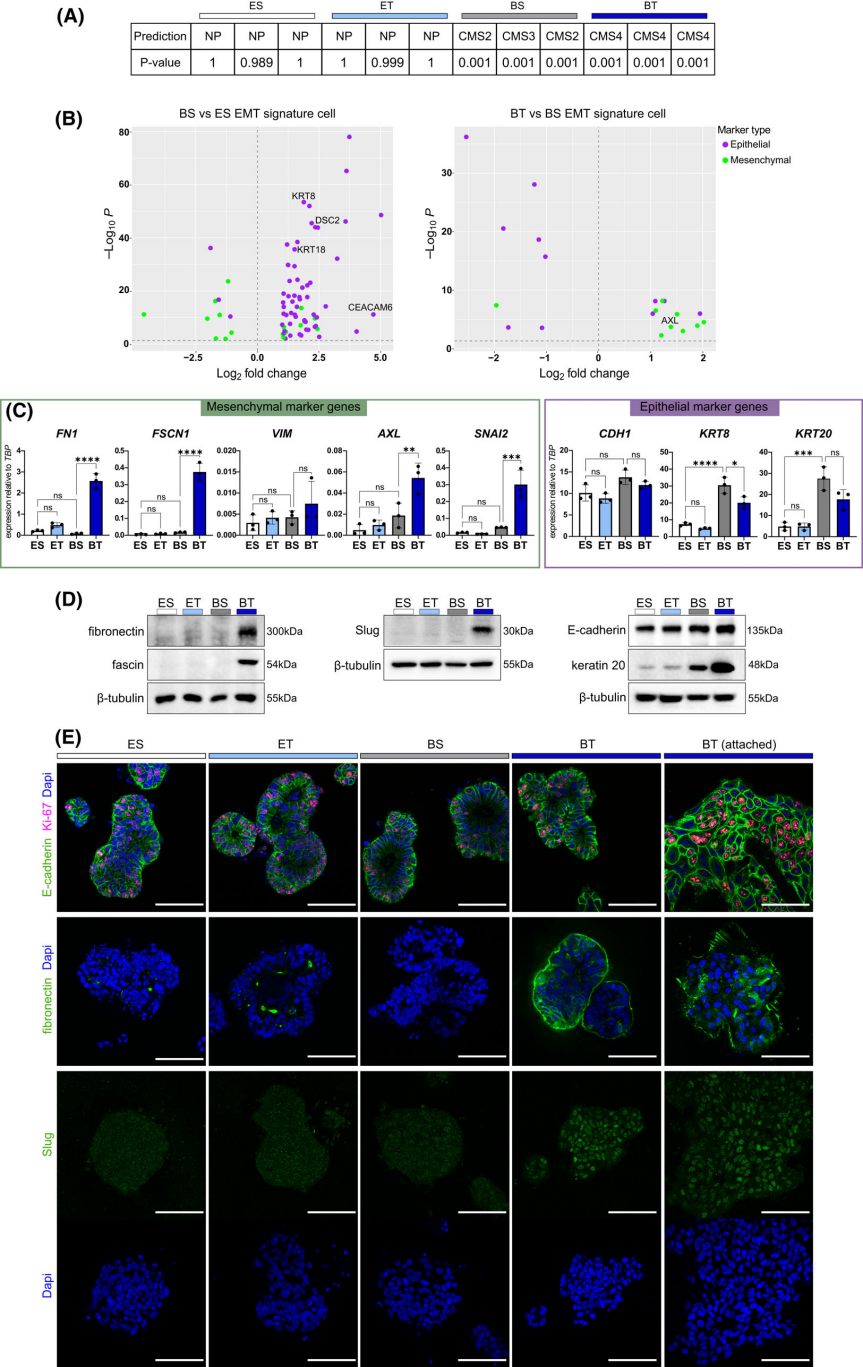
TGF‐β1 treatment of patient‐derived tumoroid 1 (PDT1) promotes differentiation towards a mesenchymal phenotype. (A) Prediction of the consensus molecular subtypes (CMS) of PDT1 cultured in different medium compositions (ES: ENAS + solvent; ET: ENAS + 5 ng·mL^−1^ TGF‐β1, BS: basal medium + solvent; BT: basal medium + 5 ng·mL^−1^ TGF‐β1) from RNA sequencing data (*n* = 3) using the CMS caller tool [34]. The predicted CMS is indicated in the first row as NP: no prediction, CMS2, CMS3 or CMS4 for each sample. The second row shows the *P*‐values for each prediction. (B) Overlap of differentially expressed genes of PDT1 cultivated for 10 days in different conditions (BS vs ES and BT vs BS) with cancer cell‐specific epithelial‐to‐mesenchymal transition (EMT) signatures [35] (*n* = 3). Volcano plot on the left represents BS versus ES, plot on the right represents BT versus BS. Epithelial genes are represented in purple and mesenchymal genes in green. (C) Gene expression analysis of mesenchymal genes (left) and epithelial genes (right) using qRT‐PCR of PDT1 cultured in different conditions as in (A). Gene expression of target genes is represented as expression relative to TATA‐Box Binding Protein (*TBP*) as housekeeping gene. Graphs show the mean and error bars depict standard deviation (SD) from three replicates (*n* = 3). Statistical significance was calculated using graphpad prism version 8 with ordinary one‐way ANOVA followed by Tukey's multiple comparison test with 95% confidence interval: ns *P* > 0.05; *P ≤ 0.05; **P ≤ 0.01; ***P ≤ 0.001; ****P ≤ 0.0001. (D) Representative western blot analysis of selected mesenchymal (fibronectin, fascin, Slug) or epithelial (E‐cadherin, keratin 20) marker proteins isolated from PDT1 treated as in (A) (*n* = 2). β‐tubulin is used as loading control. (E) Whole‐mount immunofluorescence staining and confocal microscopy of PDT1 cultured in different conditions as in (A) (*n* = 2). The top panel shows staining with antibodies against E‐cadherin as epithelial marker (green) and Ki67 as a marker for proliferative cells (red). Nuclei were counterstained with DAPI (blue). The second panel shows staining with antibodies against fibronectin as mesenchymal marker (green), and DAPI counterstain of nuclei (blue). The bottom panel shows antibody staining for Slug (green) and separate nuclear DAPI staining (blue). Scale bar: 100 μm.

To gain deeper insights into the molecular changes of the PDT1 line following TGF‐β1 treatment, we used the CMS caller tool [34] to determine the CMS, which have been used to classify CRCs into four different subtypes based on gene expression signatures [48]. While PDTs grown in ENAS conditions could not be attributed to a specific CMS subtype, PDTs grown in basal medium were classified as CMS2 (canonical) or CMS3 (metabolic) (Fig. 5A). Notably, PDTs cultivated in basal medium with TGF‐β1 were classified as CMS4, representing the mesenchymal subtype of CRC.

The top 500 upregulated genes in basal medium with TGF‐β1 compared to the ENAS condition or to basal medium without TGF‐β1 showed a significant overlap with EMT and KRAS hallmark gene sets of the MSigDB [38] (Fig. S4A,B). Moreover, the ranked DEGs of the TGF‐β1 treatment were significantly enriched for the hallmark‐EMT gene set using GSEA [37] (Fig. S4C,D).

Next, we compared gene expression profiles of this line in different media and after TGF‐β1 treatment to published EMT signature genes [35]. Cultivation of PDTs in basal medium induced the expression of epithelial marker genes, genes coding for intermediate filament‐forming keratins such as *KRT8/18*, and cell adhesion molecules including *CEACAM1* and *DSC2* compared to the ENAS‐cultured PDTs (Fig. 5B left, Table S3). Supplementation of basal medium with TGF‐β1 induced a shift from epithelial‐to‐mesenchymal gene expression signatures (Fig. 5B right, Table S3). This was also confirmed by qRT‐PCR with significant upregulation of fibronectin (*FN1*), fascin (*FSCN1*), and the receptor tyrosine kinase *AXL*, which has been associated with EMT, tumor cell invasion, and therapy resistance in different tumor entities [49] (Fig. 5C). Moreover, the expression of the EMT transcription factor *SNAI2* was significantly upregulated upon TGF‐β1 treatment. For the mesenchymal gene vimentin (*VIM*), a trend of higher expression after TGF‐β1 treatment was detectable. Epithelial marker genes including E‐cadherin (*CDH1*), *KRT8*, and *KRT20* were generally expressed at higher levels in basal medium compared to ENAS medium, confirming the differentiation phenotype of the stem cell‐like cultures. Interestingly, the addition of TGF‐β1 repressed *KRT8*, while *CDH1 and KRT20* expression remained high upon treatment. Similarly, we detected a strong protein induction of the mesenchymal markers fibronectin and fascin, as well as the *SNAI2* encoded Slug protein in basal condition following TGF‐β1 treatment, while E‐cadherin and KRT20 showed also their highest expression in this condition (Fig. 5D).

Using immunofluorescence analysis, we evaluated the protein expression of E‐cadherin, fibronectin and Slug under different conditions in the PDT1 line. Moreover, proliferation rates of PDTs were evaluated by Ki‐67 staining, which showed similar proliferation rates of PDTs in different conditions (Fig. 5E). For PDTs grown in basal medium containing TGF‐β1, we assessed both 3D structures and cells that had attached to the plates in 2D. E‐cadherin expression was detected at similar levels in all conditions (ENAS, basal medium, with/without TGF‐β1), whereas a strong induction of fibronectin and Slug was apparent only in PDTs cultured in basal medium after TGF‐β1 treatment. Importantly, cells that grew in 2D showed prominent fibril formation, a key process in TGF‐β induced EMT [50].

Collectively, these results indicate that treatment with TGF‐β1 shifts the differentiation of PDTs towards a mesenchymal phenotype, showing gene expression characteristics of partial EMT characterized by simultaneous expression of epithelial and mesenchymal marker genes.

### 3.5 TGF‐β1 induces invasive properties in CRC PDT1

**Fig. 6:**
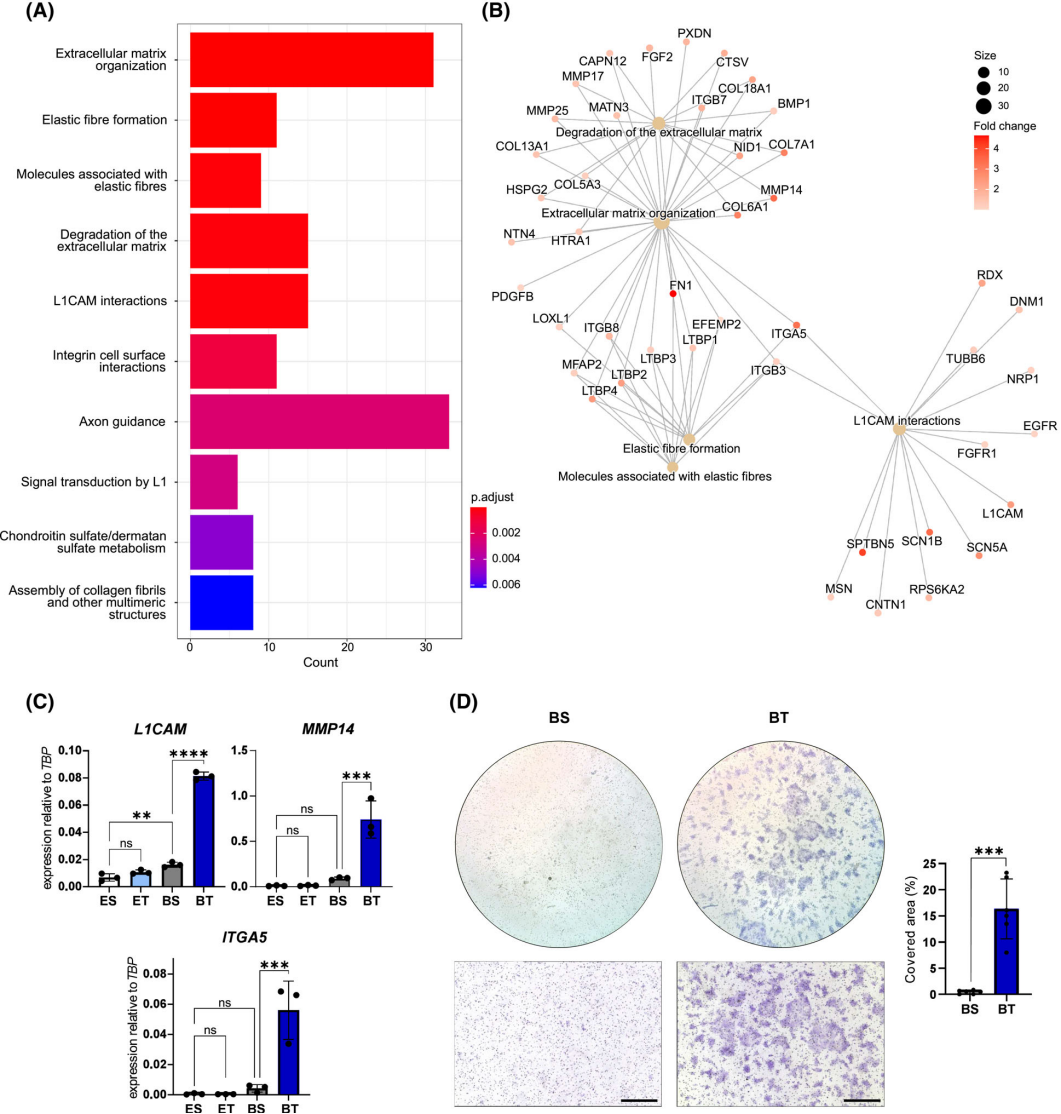
TGF‐β1 induces invasive properties in patient‐derived tumoroid 1 (PDT1). (A) Bar chart representing the top significantly enriched pathways in BT (basal medium + TGF‐β1) versus BS (basal medium + solvent) conditions, based on Reactome pathway analysis of significantly upregulated genes (*P*adj < 0.05 and log_2_ fold change (LFC) > 1) from RNA sequencing analysis between the two conditions (*n* = 3). X‐axis represents the counts of individual genes upregulated in the respective pathway. Color gradient indicates significance of the enriched pathway based on *P*adj. (B) CNET (Gene‐Concept Network)‐plot representing connections of genes among the five top‐ enriched Reactome pathways (according to *P*adj) from (A). Circle size indicates gene count for each pathway. Dot color for each gene indicates LFC of the gene according to the color gradient. (C) qRT‐PCR analysis of genes associated with invasion (*L1CAM*, *MMP14*, *ITGA5*) relative to the reference gene TATA‐Box Binding Protein (*TBP*) of PDT1 cultured in different conditions (ES: ENAS + solvent; ET: ENAS + 5 ng·mL^−1^ TGF‐β1, BS: basal medium + solvent; BT: basal medium + 5 ng·mL^−1^ TGF‐β1). Graphs show the mean and standard deviation (SD) from three replicates (*n* = 3). Statistical significance was calculated with graphpad prism version 8 using ordinary one‐way ANOVA followed by Tukey's multiple comparison test with 95% confidence interval: ns *P* > 0.05; **P ≤ 0.01; ***P ≤ 0.001; ****P ≤ 0.0001. (D) Representative bright‐field images of trans‐well invasion assays in basal medium + solvent (BS) or in basal medium + 5 ng·mL^−1^ TGF‐β1 (BT) towards fetal calf serum (FCS) as chemoattractant (*n* = 3). Invasive cells were visualized by crystal violet staining. Overview (top) and higher magnification image (bottom) are shown. Please note that bottom panels are not enlargements of the top panels. Scale bar: 500 μm (lower panel). Right panel shows quantification of invasive cells, depicted as percent covered area. The graph represents the mean and standard deviation (SD) of three counted areas from two individual experiments per condition (*n* = 2). Statistical significance was calculated with graphpad prism version 8 using Welch's *t*‐test: ***P ≤ 0.001.

Next, Reactome pathway analysis [32] was employed on significantly DEGs between PDT1 cultured in basal medium with or without TGF‐β1. Notably, we observed enrichment of pathways associated with ECM organization/degradation, elastic fiber formation, and cell adhesion (Fig. 6A, Table S4). Within these pathways, several cell adhesion molecules, including integrins (*ITGB3,7,8*), latent TGF‐β binding proteins (*LTBP1‐4*), and *L1CAM‐*associated molecules, as well as ECM‐associated proteins such as collagens, fibronectin, and metalloproteinases (*MMP14,17*), were among the top deregulated genes (Fig. 6B). The upregulation of the transmembrane protein *L1CAM*, which is highly expressed in metastasis initiating cells in CRC [51], *MMP14* and *ITGA5*, which represent key molecules for ECM reorganization and elastic fiber formation, was additionally confirmed by qRT‐PCR (Fig. 6C). Collectively, these findings suggest substantial alterations in the mechano‐chemical properties of PDTs grown under TGF‐β1 conditions, probably enhancing their invasive potential. Thus, Matrigel trans‐well invasion assays were performed to investigate the invasive properties of PDT1 under TGF‐β1 exposure. While PDTs cultured in basal medium exhibited no invasive potential, TGF‐β1 treatment induced high invasive capacities of PDTs, represented by substantial migration of tumor cells through the pores of the trans‐well mesh (Fig. 6D).

In conclusion, TGF‐β1 treatment significantly upregulates pathways involved in ECM remodeling and partial EMT characteristics of PDTs *in vitro*, thereby prominently enhancing their invasive properties.

## 4 Discussion

Classical TGF‐β studies utilized cell lines grown in 2D, which do not faithfully replicate several aspects important for EMT such as invasive properties, cell morphology and polarity, or cell‐cell and cell‐ECM interactions [52, 53, 54]. The advent of innovative organoid and tumoroid models has provided suitable tools and novel insights into the effects of TGF‐β on healthy and malignant cells [4, 13, 18, 55, 56]. However, research on CRC tumoroids delivered partially inconclusive findings regarding the potential mechanisms underlying the TGF‐β duality in CRC. Normal colon organoids, adenoma‐derived organoids, as well as genetically engineered *BRAF* ^V600E^ organoids, displayed phenotypic and transcriptional changes, indicating a critical importance of TGF‐β already in precursor lesions directing specific adenoma subtypes to the aggressive mesenchymal CMS4 CRC subtype [55]. Studies on human tumoroids showed divergent results, demonstrating either a tumor‐suppressive response and minimal changes in EMT marker gene expression [4] or *KRAS*‐dependent resistance to TGF‐β treatment through downregulation of the pro‐apoptotic protein BIM [13]. Our study included five primary CRC PDTs with intact TGF‐β signaling and different *KRAS* mutations. Notably, only one of the five lines, which harbored an atypical *KRAS* ^Q22K^ mutation (PDT1), maintained proliferation and showed phenotypic changes, while pharmacological KRAS inhibition rendered the line highly sensitive to TGF‐β induced cell death. The *KRAS* ^Q22K^ was previously associated with a 300‐fold higher activity for inducing ERK *in vitro* compared to the frequently occurring exon 2 *KRAS* ^G13D^ mutation [57]. Its rare occurrence in about 0.2% of CRCs might suggest a selection against MAPK/ERK hyperactivation, which is also reflected in the mutual exclusive occurrence of BRAF^V600E^ and KRAS^G12D^ mutations in different cancer entities [58]. The remaining four *SMAD4* wild‐type primary PDTs harbored *KRAS* ^G12D^ (PDT2,5), *KRAS* ^G12V^ (PDT4) or exon 4 *KRAS* ^A146T^ (PDT3) mutations and exhibited tumor‐suppressive effects upon TGF‐β1 treatment. Importantly, the different *KRAS* mutations have been associated with diverse treatment responses to chemotherapy and tyrosine kinase receptor‐targeting therapy, such as EGFR therapy, as well as patient outcome [59]. Our data suggests that individual *KRAS* mutations result in different activation levels of MAPK signaling via phospho‐ERK1, causing a disbalance of pro‐ and anti‐apoptotic signaling via BIM and Bcl‐xL. Thus, the *KRAS* mutation status might be directly linked to TGF‐β1 responses, impacting on tumor aggressiveness and therapy response of patients.

Organoids and tumoroids are classically cultured in media promoting their stem cell properties [20]. However, since TGF‐β signaling is highly context dependent, we here utilized an adapted medium, which only contained EGF as a growth factor and omitted classical ENAS medium factors and TGF‐β pathway inhibitors for PDT cultivation. As expected, this resulted in downregulation of stem cell genes and upregulation of genes associated with specific colon cell subtypes including enterocytes and various secretory cell types. We previously observed similar phenotypes of PDTs upon co‐culture with CAFs, which secrete several growth factors and cytokines, enabling the growth of tumoroids without the addition of niche factors [19]. The observed diversity in colonic cell subtypes in this co‐culture system resembled the *in vivo* tumors to a high degree [19]. Therefore, we suggest that media allowing for the differentiation of PDTs recapitulate *in vivo* tumor characteristics better than the commonly used ENAS medium and are thus more suitable to study TGF‐β responses. The critical assessment of media conditions was also highlighted in a recent report on the drug response of PDTs derived from high‐grade serous ovarian cancer [60].

Besides the upregulation of mesenchymal markers, the PDT1 line retained some epithelial characteristics following TGF‐β1 treatment. This is reminiscent of partial EMT, which has been described for several cancer entities including breast, lung, and CRC, and is characterized by a heterogeneous population of tumor cells in different states of EMT [61]. Notably, tumors displaying this incomplete acquisition of mesenchymal features possess the highest metastatic potential [61]. Along these lines, we observed deregulated expression of genes and pathways involved in ECM organization/degradation, elastic fiber formation, and cell adhesion, which was paralleled by significantly increased invasive properties of tumoroid cells upon TGF‐β1 stimulation. Moreover, some marker genes including the TF *PAX6* were specifically upregulated in basal medium following TGF‐β1 treatment. An oncogenic role for PAX6 was previously reported for different cancer entities including CRC, breast, and non‐small cell lung cancer [62, 63, 64]. *PAX6* gene expression is regulated by canonical TGF‐β1 signaling through SMAD3 binding to its promoter region [65]. Moreover, PAX6 can interact with the MHC1 domain of different SMADs including SMAD3 and SMAD1 [66], suggesting that PAX6 represents a context‐dependent transcription factor for CRC progression and metastasis, in line with the observed EMT phenotype. Together, these findings suggest that tumor cells harbor intrinsic capabilities to remodel the ECM and allow increased tumor cell motility, which was previously suggested to depend largely on stromal fibroblasts in the TME [67].

## 5 Conclusion

In conclusion, our findings show that context‐dependent effects of TGF‐β can be replicated *in vitro* in CRC PDT models, and most likely depend on the presence of aggressive *KRAS* mutations, restricting pro‐apoptotic signaling. Our findings underline the tumor cell‐specific effects of TGF‐β for the induction of a partial EMT state, matrix remodeling, and invasion. Moreover, our data adds important aspects to the relevance of the *KRAS* gene mutation spectrum for tumor progression and metastasis, which has the potential to impact future research directions and therapeutic strategies in personalized medicine.

## Conflict of interest

The authors declare no conflict of interest.

## Author contributions

GE acquired funding and supervised the project. TM and GE designed the project and wrote the manuscript. TM, JeK, PK, MM, PMS, AB performed and analyzed experiments. LT established PDT cultures and protocols. TM, CUPM, KD and RS‐T performed data analysis and visualization. JP, JuK, VSA provided PDT lines. MB provided patient material and clinical data. JeK helped with writing and scientific discussion. AT performed pathological assessment. LW‐O and LM carried out PDT panel sequencing.

## Peer review

The peer review history for this article is available at https://www.webofscience.com/api/gateway/wos/peer‐review/10.1002/1878‐0261.70014.

## Supporting information


**Fig. S1.** TGF‐β1 treatment induces morphological changes and 2D growth in patient‐derived tumoroid 1 (PDT1).
**Fig. S2.** TGF‐β1 enhances the sensitivity of patient‐derived tumoroid 1 (PDT1) towards KRAS inhibition.
**Fig. S3.1.** Cultivation of patient‐derived tumoroid 1 (PDT1) in basal medium stimulates differentiation towards specialized cell types of the colon crypt.
**Fig. S3.2.** Cultivation of patient‐derived tumoroid 1 (PDT1) in basal medium stimulates differentiation towards specialized cell types of the colon crypt.
**Fig. S4.** Gene overlap and Gene set enrichment analysis (GSEA) analysis of TGF‐β1 induced genes.
**Table S1.** Excel file containing significant deregulated genes between different conditions.
**Table S2.** Excel file containing gene lists of different cell types of the colon crypt, related to Fig. 4F and S3.1.
**Table S3.** Excel file containing epithelial‐to‐mesenchymal transition (EMT) genes shown in Fig. 5B.
**Table S4.** Excel file containing top significant up‐ and downregulated Reactome pathways and associated genes, related to Fig. 6A.

## Data Availability

The analysis of the RNA sequencing data is available within the results section and the supplementary information files. The RNA sequencing raw data are publicly available at Gene Expression Omnibus GSE270815.
