# Supplementary material for: The atypical KRAS Q22K mutation directs TGF‐β response towards partial epithelial‐to‐mesenchymal transition in patient‐derived colorectal cancer tumoroids
Source: Mol Oncol. 2025 Mar 11;19(8):2212–32. doi: 10.1002/1878-0261.70014 (PMC12330932; doi:10.1002/1878-0261.70014)
Supplement: Supplementary file 1 — Fig. S1. TGF‐β1 treatment induces morphological changes and 2D growth in patient‐derived tumoroid 1 (PDT1). Fig. S2. TGF‐β1 enhances the sensitivity of patient‐derived tumoroid 1 (PDT1) towards KRAS inhibition. Fig. S3.1. Cultivation of patient‐derived tumoroid 1 (PDT1) in basal medium stimulates differentiation towards specialized cell types of the colon crypt. Fig. S3.2. Cultivation of patient‐derived tumoroid 1 (PDT1) in basal medium stimulates differentiation towards specialized cell types of the colon crypt. Fig. S4. Gene overlap and Gene set enrichment analysis (GSEA) analysis of TGF‐β1 induced genes. Table S1. Excel file containing significant deregulated genes between different conditions. Table S2. Excel file containing gene lists of different cell types of the colon crypt, related to Fig. 4F and S3.1. Table S3. Excel file containing epithelial‐to‐mesenchymal transition (EMT) genes shown in Fig. 5B. Table S4. Excel file containing top significant up‐ and downregulated Reactome pathways and associated genes, related to Fig. 6A. [file MOL2-19-2212-s001.zip › mol270014-sup-0003-FigureS3.1.pdf]

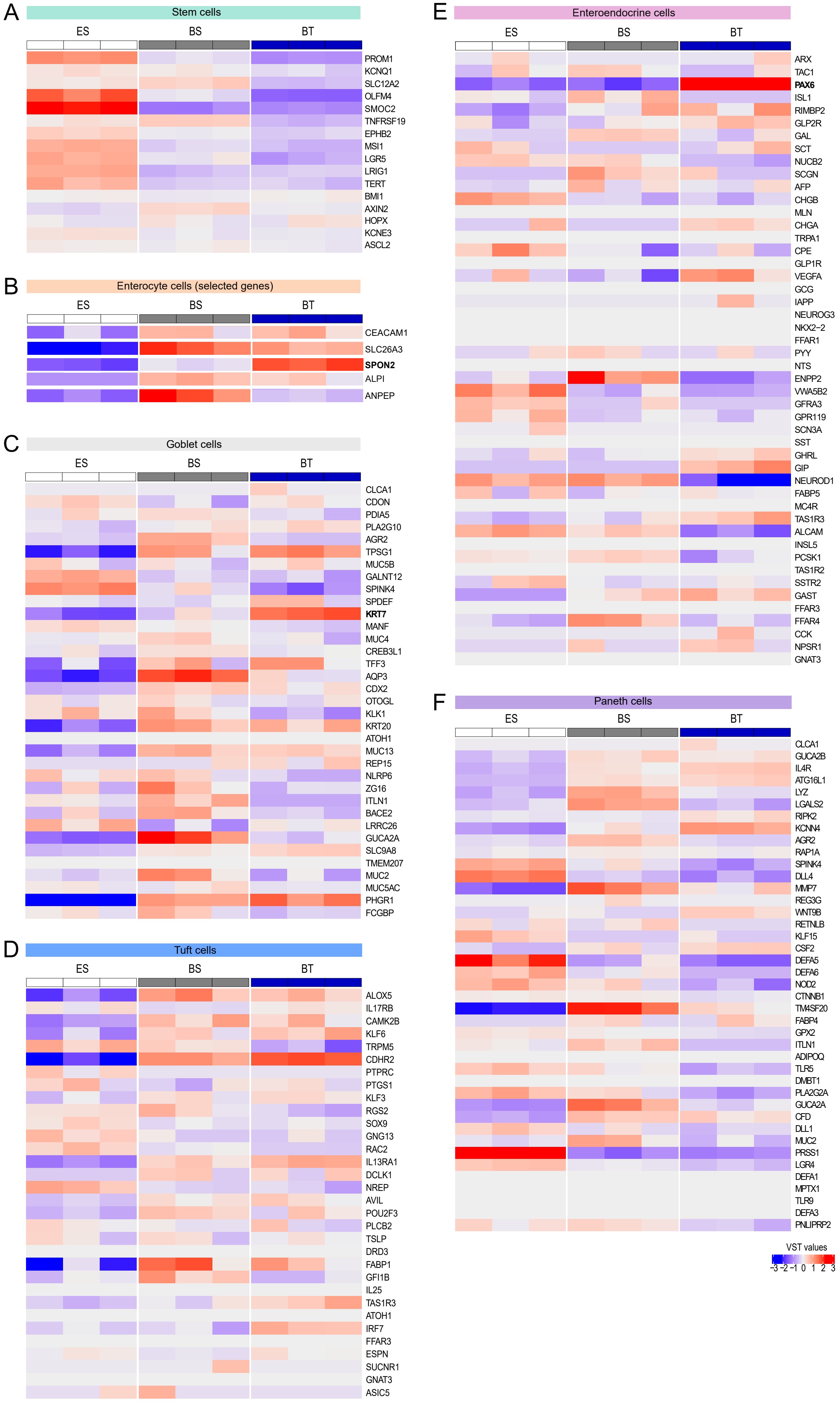
A
Stem cells
ES
BS
BT
PROM1
KCNQ1
SLC12A2
OLFM4
SMOC2
TNFRSF19
EPHB2
MSI1
LGR5
LRIG1
TERT
BMI1
AXIN2
HOPX
KCNE3
ASCL2
B
Enterocyte cells (selected genes)
ES
BS
BT
CEACAM1
SLC26A3
SPON2
ALPI
ANPEP
C
Goblet cells
ES
BS
BT
CLCA1
CDON
PDIA5
PLA2G10
AGR2
TPSG1
MUC5B
GALNT12
SPINK4
SPDEF
KRT7
MANF
MUC4
CREB3L1
TFF3
AQP3
CDX2
OTOGL
KLK1
KRT20
ATOH1
MUC13
REP15
NLRP6
ZG16
ITLN1
BACE2
LRRC26
GUCA2A
SLC9A8
TMEM207
MUC2
MUC5AC
PHGR1
FCGBP
D
Tuft cells
ES
BS
BT
ALOX5
IL17RB
CAMK2B
KLF6
TRPM5
CDHR2
PTPRC
PTGS1
KLF3
RGS2
SOX9
GNG13
RAC2
IL13RA1
DCLK1
NREP
AVIL
POU2F3
PLCB2
TSLP
DRD3
FABP1
GFI1B
IL25
TAS1R3
ATOH1
IRF7
FFAR3
ESPN
SUCNR1
GNAT3
ASIC5
E
Enteroendocrine cells
ES
BS
BT
ARX
TAC1
PAX6
ISL1
RIMBP2
GLP2R
GAL
SCT
NUCB2
SCGN
AFP
CHGB
MLN
CHGA
TRPA1
CPE
GLP1R
VEGFA
GCG
IAPP
NEUROG3
NKX2−2
FFAR1
PYY
NTS
ENPP2
VWA5B2
GFRA3
GPR119
SCN3A
SST
GHRL
GIP
NEUROD1
FABP5
MC4R
TAS1R3
ALCAM
INSL5
PCSK1
TAS1R2
SSTR2
GAST
FFAR3
FFAR4
CCK
NPSR1
GNAT3
F
Paneth cells
ES
BS
BT
CLCA1
GUCA2B
IL4R
ATG16L1
LYZ
LGALS2
RIPK2
KCNN4
AGR2
RAP1A
SPINK4
DLL4
MMP7
REG3G
WNT9B
RETNLB
KLF15
CSF2
DEFA5
DEFA6
NOD2
CTNNB1
TM4SF20
FABP4
GPX2
ITLN1
ADIPOQ
TLR5
DMBT1
PLA2G2A
GUCA2A
CFD
DLL1
MUC2
PRSS1
LGR4
DEFA1
MPTX1
TLR9
DEFA3
PNLIPRP2
VST values
−3 −2 −1 0 1 2 3
