# Supplementary material for: The atypical KRAS Q22K mutation directs TGF‐β response towards partial epithelial‐to‐mesenchymal transition in patient‐derived colorectal cancer tumoroids
Source: Mol Oncol. 2025 Mar 11;19(8):2212–32. doi: 10.1002/1878-0261.70014 (PMC12330932; doi:10.1002/1878-0261.70014)
Supplement: Supplementary file 1 — Fig. S1. TGF‐β1 treatment induces morphological changes and 2D growth in patient‐derived tumoroid 1 (PDT1). Fig. S2. TGF‐β1 enhances the sensitivity of patient‐derived tumoroid 1 (PDT1) towards KRAS inhibition. Fig. S3.1. Cultivation of patient‐derived tumoroid 1 (PDT1) in basal medium stimulates differentiation towards specialized cell types of the colon crypt. Fig. S3.2. Cultivation of patient‐derived tumoroid 1 (PDT1) in basal medium stimulates differentiation towards specialized cell types of the colon crypt. Fig. S4. Gene overlap and Gene set enrichment analysis (GSEA) analysis of TGF‐β1 induced genes. Table S1. Excel file containing significant deregulated genes between different conditions. Table S2. Excel file containing gene lists of different cell types of the colon crypt, related to Fig. 4F and S3.1. Table S3. Excel file containing epithelial‐to‐mesenchymal transition (EMT) genes shown in Fig. 5B. Table S4. Excel file containing top significant up‐ and downregulated Reactome pathways and associated genes, related to Fig. 6A. [file MOL2-19-2212-s001.zip › Supporting_Information2_1.docx]

**Supporting information**

**Figure S1: TGF-β1 treatment induces morphological changes and 2D growth in patient-derived tumoroid 1 (PDT1).**

Bar chart representing the quantification of the percentage of 2D growth patterns of PDT1 cultivated in the different conditions (ES: ENAS + solvent; ET: ENAS + 5 ng/ml TGF-β1; BS: basal medium + solvent; BT: basal medium + 5 ng/ml TGF-β1) for 10 days (n=3). Percentage is shown as 2D/3D representing mean values with standard deviation (SD) indicated by error bars. Statistical significance was calculated with GraphPad Prism version 8 using ordinary one-way ANOVA followed by Tukey’s multiple comparison test with 95% confidence interval: ns P > 0.05; *P ≤ 0.05; ****P ≤ 0.0001.

**Figure S2: TGF-β1 enhances the sensitivity of patient-derived tumoroid 1 (PDT1) towards KRAS inhibition.**

Dose response curves of PDT1 exposed to BI-2865 (up to 50 µM) (**A**) or ACBI3 (up to 30 µM) (**B**) indicating viability (% of solvent control) of PDT1 in relation to increasing drug doses. Curves were fitted using non-linear regression. Data shows mean with error bars depicting standard deviation (SD) of three technical replicates**. C** Representative bright-field microscopic images of PDT1 cultured in basal medium plus solvent (BS) or basal medium plus TGF-β1 (5 ng/ml) (BT) untreated or treated with KRAS inhibitors (BI-2865: 0.37 µM; ACBI3: 0.12 µM) (n=2). **D** Cell viability of PDT1 in basal media treated or untreated with TGF-β1 or KRAS inhibitors (BI-2865, ACBI3) measured with CellTiter-Glo® 3D Cell Viability Assay. Viability is presented as % of viability relative to basal medium with solvent (BS) (n=2), with three technical replicates each). Values are represented as mean with standard deviation (SD) indicated by error bars. Statistical significance was calculated with GraphPad Prism version 8 using ordinary one-way ANOVA followed by Tukey’s multiple comparison test with 95% confidence interval: *P ≤ 0.05; ***P ≤ 0.001.

**Figure S3.1:** **Cultivation of patient-derived tumoroid 1 (PDT1) in basal medium stimulates differentiation towards specialized cell types of the colon crypt.**

Heatmaps showing variance stabilizing transformation (VST) gene count values of PDT1 cultured for 10 days in different conditions, ES: ENAS + solvent; BS: basal medium + solvent; BT: basal medium + 5 ng/ml TGF-β1 for the cell type-associated genes inferred from (<https://panglaodb.se/>) for stem cells (**A**), enterocyte cells (**B**), goblet cells (**C**), tuft cells (**D**), enteroendocrine cells (**E**) and Paneth cells **F**). Red indicates upregulation, blue downregulation (n=3).

**Figure S3.2:** **Cultivation of patient-derived tumoroid 1 (PDT1) in basal medium stimulates differentiation towards specialized cell types of the colon crypt.**

**A** Western blot analysis of PDT1 cultivated in the different conditions for 10 days: ES: ENAS + solvent; BS: basal medium + solvent; BT: basal medium + 5 ng/ml TGF-β1 using antibodies for the stem cell marker LGR5 (* indicates unspecific band), the enterocyte cell markers ANPEP and CEACAM1 and the enteroendocrine cell marker PAX6. β-tubulin and β-actin represent loading controls (n=2). LGR5 and ANPEP were sequentially probed on the same membrane and thus show the identical loading control for ß-tubulin. The enteroendocrine marker PAX6 was sequentially probed with the EMT marker Slug (shown in main Figure 5D) and therefore shows the identical ß-tubulin control. **B** Whole-mount immunofluorescence staining and confocal microscopy of PDT1 cultured in different conditions as in (A) (n=2). The top panel shows nuclear staining against PAX6 as enteroendocrine marker (green) counterstained with DAPI (blue). Single channel pictures for PAX6 staining are shown below the multi-channel picture in black and white. Scale bar: 100 µm.

**Figure S4: Gene overlap and Gene set enrichment analysis (GSEA) analysis of TGF-β1 induced genes.**

Bubble chart illustrating the top 10 significant Molecular Signatures Database (MSigDB) Hallmark pathways derived from gene overlap analysis of the top 500 significantly overexpressed genes in BT (basal medium + 5 ng/ml TGF-β1) versus ES (ENAS + solvent) conditions (**A**) or BT versus BS (basal + solvent) conditions (**B**) using the GSEA browser tool (<https://www.gsea-msigdb.org>) (n=3). Pathways are ranked by significance based on false discovery rate (FDR q-value). Size of the bubbles represents number of genes in the pathway that were overexpressed in the BT condition. **C** GSEA of significantly differentially expressed genes comparing BT versus ES, or BT versus BS (**D**) (n=3).

**Table S1.** Excel file containing significant deregulated genes between different conditions.

**Table S2.** Excel file containing gene lists of different cell types of the colon crypt, related to **Figure 4F** and **S3.1**.

**Table S3.** Excel file containing epithelial-to-mesenchymal transition (EMT) genes shown in **Figure 5B**.

**Table S4.** Excel file containing top significant up- and down-regulated Reactome pathways and associated genes, related to **Figure 6A**.
