# Supplementary figures and images for: The atypical KRAS Q22K mutation directs TGF‐β response towards partial epithelial‐to‐mesenchymal transition in patient‐derived colorectal cancer tumoroids
Source: Mol Oncol. 2025 Mar 11;19(8):2212–32. doi: 10.1002/1878-0261.70014 (PMC12330932; doi:10.1002/1878-0261.70014)

Percentage of 2D tumoroids  
PDT1

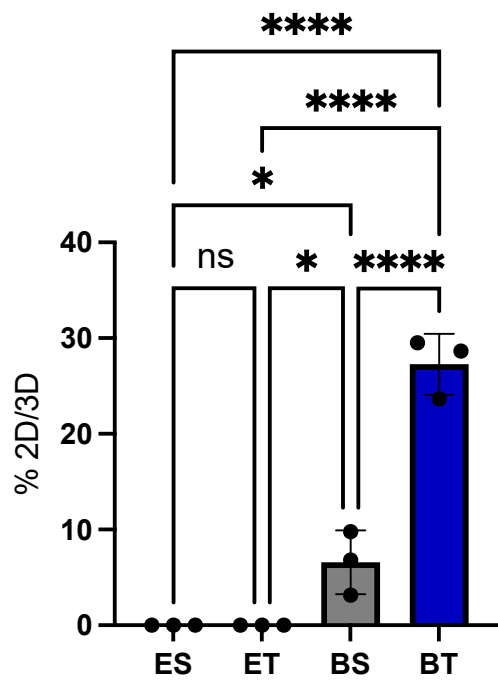

Supplement: Supplementary file 1 — Fig. S1. TGF‐β1 treatment induces morphological changes and 2D growth in patient‐derived tumoroid 1 (PDT1). Fig. S2. TGF‐β1 enhances the sensitivity of patient‐derived tumoroid 1 (PDT1) towards KRAS inhibition. Fig. S3.1. Cultivation of patient‐derived tumoroid 1 (PDT1) in basal medium stimulates differentiation towards specialized cell types of the colon crypt. Fig. S3.2. Cultivation of patient‐derived tumoroid 1 (PDT1) in basal medium stimulates differentiation towards specialized cell types of the colon crypt. Fig. S4. Gene overlap and Gene set enrichment analysis (GSEA) analysis of TGF‐β1 induced genes. Table S1. Excel file containing significant deregulated genes between different conditions. Table S2. Excel file containing gene lists of different cell types of the colon crypt, related to Fig. 4F and S3.1. Table S3. Excel file containing epithelial‐to‐mesenchymal transition (EMT) genes shown in Fig. 5B. Table S4. Excel file containing top significant up‐ and downregulated Reactome pathways and associated genes, related to Fig. 6A. [file MOL2-19-2212-s001.zip › mol270014-sup-0001-FigureS1.pdf]

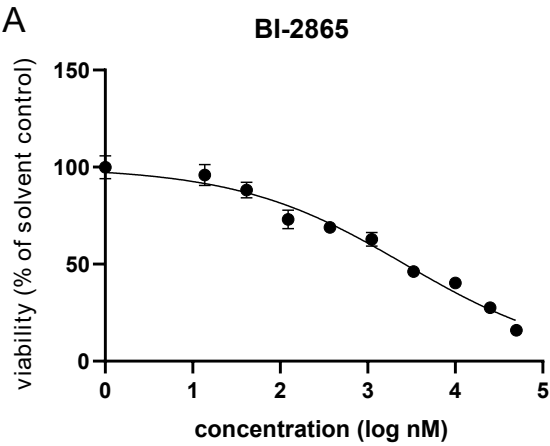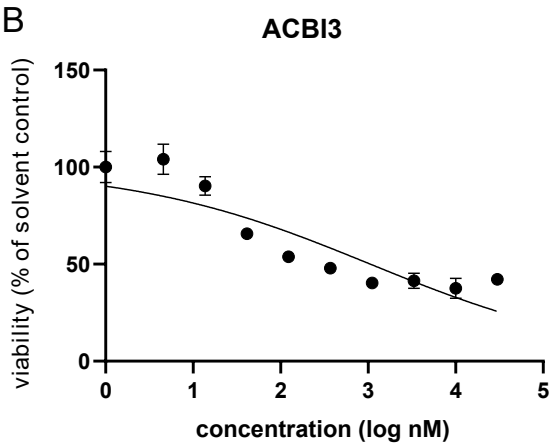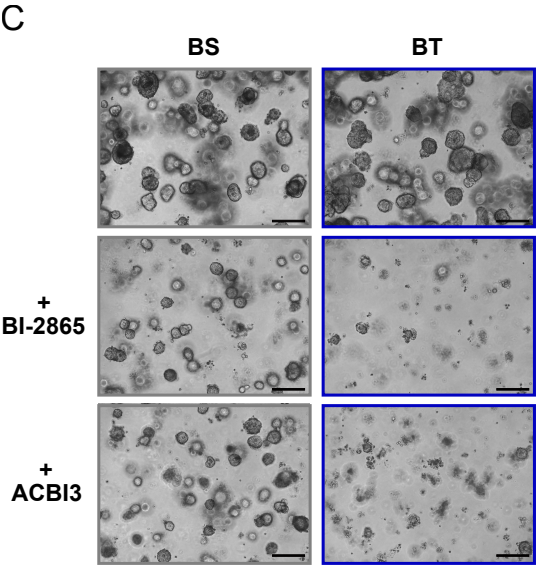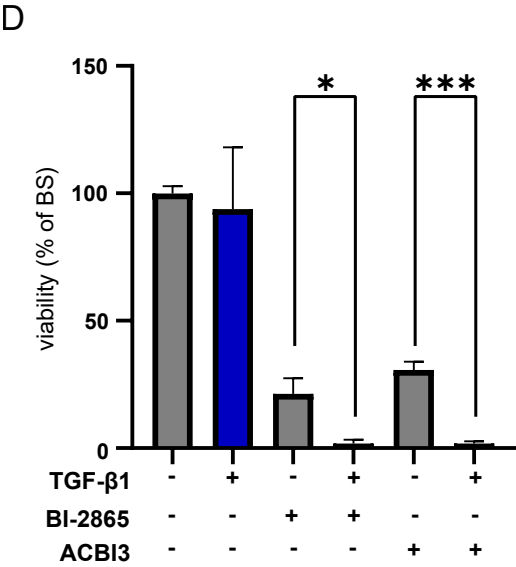

Supplement: Supplementary file 1 — Fig. S1. TGF‐β1 treatment induces morphological changes and 2D growth in patient‐derived tumoroid 1 (PDT1). Fig. S2. TGF‐β1 enhances the sensitivity of patient‐derived tumoroid 1 (PDT1) towards KRAS inhibition. Fig. S3.1. Cultivation of patient‐derived tumoroid 1 (PDT1) in basal medium stimulates differentiation towards specialized cell types of the colon crypt. Fig. S3.2. Cultivation of patient‐derived tumoroid 1 (PDT1) in basal medium stimulates differentiation towards specialized cell types of the colon crypt. Fig. S4. Gene overlap and Gene set enrichment analysis (GSEA) analysis of TGF‐β1 induced genes. Table S1. Excel file containing significant deregulated genes between different conditions. Table S2. Excel file containing gene lists of different cell types of the colon crypt, related to Fig. 4F and S3.1. Table S3. Excel file containing epithelial‐to‐mesenchymal transition (EMT) genes shown in Fig. 5B. Table S4. Excel file containing top significant up‐ and downregulated Reactome pathways and associated genes, related to Fig. 6A. [file MOL2-19-2212-s001.zip › mol270014-sup-0002-FigureS2.pdf]

A

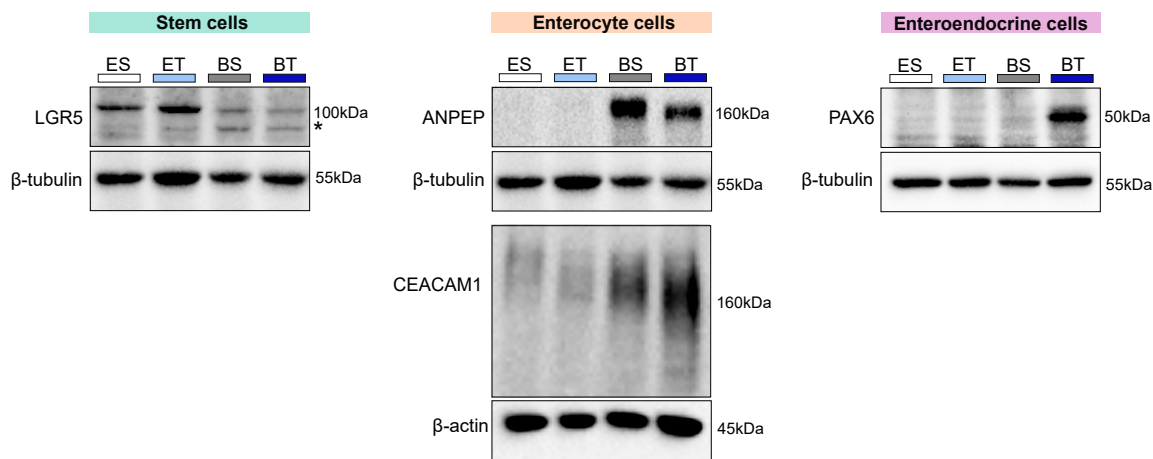

B

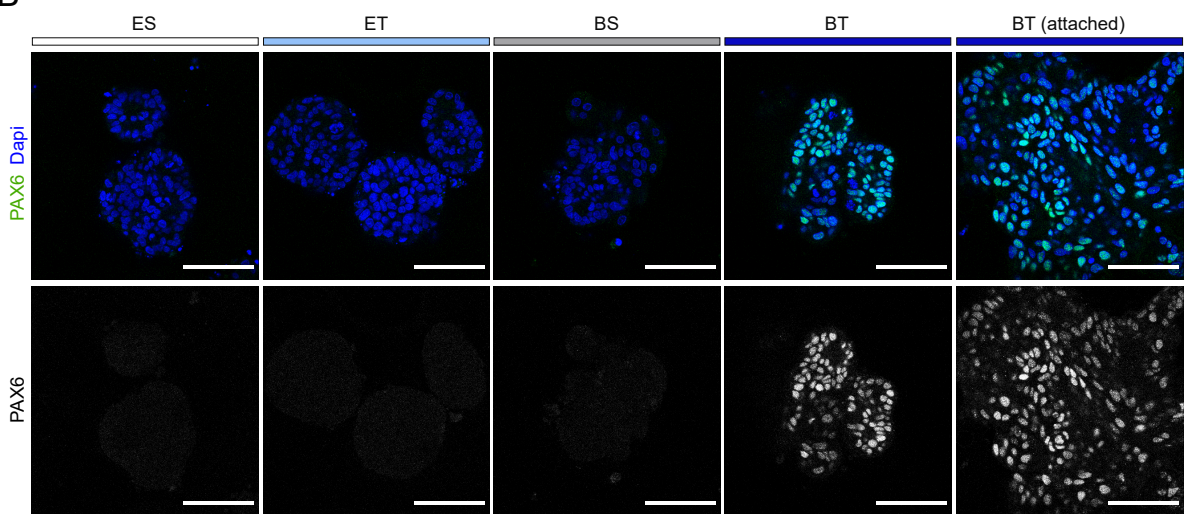

Supplement: Supplementary file 1 — Fig. S1. TGF‐β1 treatment induces morphological changes and 2D growth in patient‐derived tumoroid 1 (PDT1). Fig. S2. TGF‐β1 enhances the sensitivity of patient‐derived tumoroid 1 (PDT1) towards KRAS inhibition. Fig. S3.1. Cultivation of patient‐derived tumoroid 1 (PDT1) in basal medium stimulates differentiation towards specialized cell types of the colon crypt. Fig. S3.2. Cultivation of patient‐derived tumoroid 1 (PDT1) in basal medium stimulates differentiation towards specialized cell types of the colon crypt. Fig. S4. Gene overlap and Gene set enrichment analysis (GSEA) analysis of TGF‐β1 induced genes. Table S1. Excel file containing significant deregulated genes between different conditions. Table S2. Excel file containing gene lists of different cell types of the colon crypt, related to Fig. 4F and S3.1. Table S3. Excel file containing epithelial‐to‐mesenchymal transition (EMT) genes shown in Fig. 5B. Table S4. Excel file containing top significant up‐ and downregulated Reactome pathways and associated genes, related to Fig. 6A. [file MOL2-19-2212-s001.zip › mol270014-sup-0004-FigureS3.2.pdf]

A

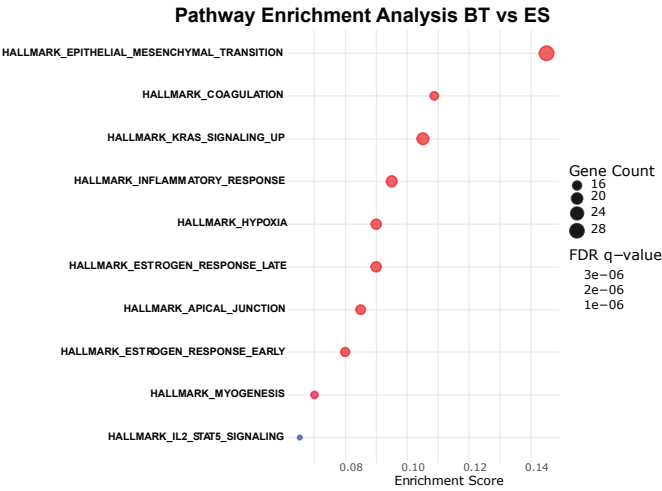

B

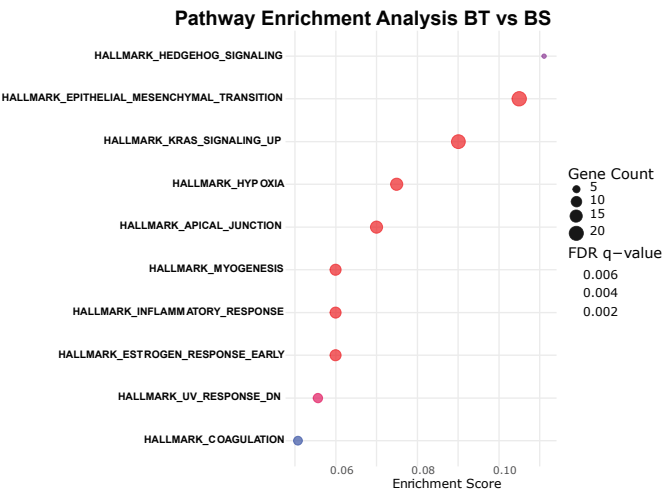

C

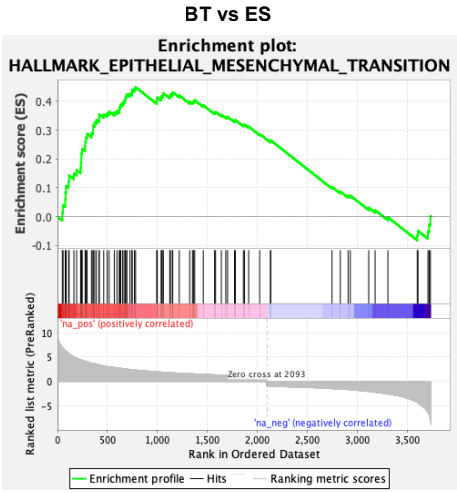

D

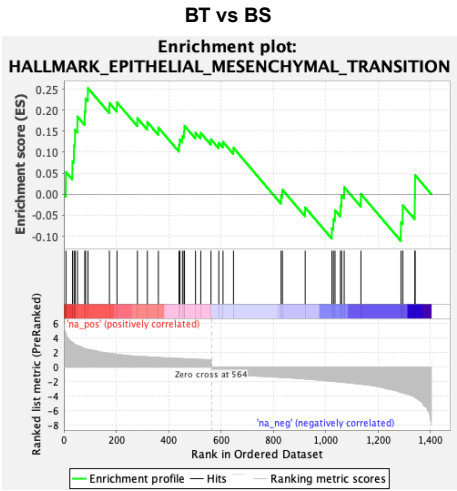

Supplement: Supplementary file 1 — Fig. S1. TGF‐β1 treatment induces morphological changes and 2D growth in patient‐derived tumoroid 1 (PDT1). Fig. S2. TGF‐β1 enhances the sensitivity of patient‐derived tumoroid 1 (PDT1) towards KRAS inhibition. Fig. S3.1. Cultivation of patient‐derived tumoroid 1 (PDT1) in basal medium stimulates differentiation towards specialized cell types of the colon crypt. Fig. S3.2. Cultivation of patient‐derived tumoroid 1 (PDT1) in basal medium stimulates differentiation towards specialized cell types of the colon crypt. Fig. S4. Gene overlap and Gene set enrichment analysis (GSEA) analysis of TGF‐β1 induced genes. Table S1. Excel file containing significant deregulated genes between different conditions. Table S2. Excel file containing gene lists of different cell types of the colon crypt, related to Fig. 4F and S3.1. Table S3. Excel file containing epithelial‐to‐mesenchymal transition (EMT) genes shown in Fig. 5B. Table S4. Excel file containing top significant up‐ and downregulated Reactome pathways and associated genes, related to Fig. 6A. [file MOL2-19-2212-s001.zip › mol270014-sup-0005-FigureS4.pdf]
